# Emotional Eating Interventions for Adults Living with Overweight or Obesity: A Systematic Review and Meta-Analysis

**DOI:** 10.3390/ijerph20032722

**Published:** 2023-02-03

**Authors:** Jo Smith, Xiao Qi Ang, Emma L. Giles, Gemma Traviss-Turner

**Affiliations:** 1 School of Health and Life Sciences, Teesside University, Middlesbrough TS1 3BX, UK; 2 Tees, Esk and Wear Valleys NHS Foundation Trust, Darlington DL2 2TS, UK; 3 Leeds Institute of Health Sciences, University of Leeds, Leeds LS2 9JT, UK

**Keywords:** emotional eating, obesity, weight, behaviour, mindfulness, systematic review

## Abstract

Background: Emotional eating (EE) may be defined as a tendency to eat in response to negative emotions and energy-dense and palatable foods, and is common amongst adults with overweight or obesity. There is limited evidence regarding the effectiveness of interventions that address EE. Objectives: To synthesize evidence on the effectiveness of EE interventions for weight loss and EE in adults living with overweight or obesity. Methods: This is a systematic review and meta-analysis. Adhering to the PRISMA guidance, a comprehensive electronic search was completed up to February 2022. Random effects meta-analysis was carried out to determine the percentage change in weight and EE scores. Results: Thirty-four studies were included. The combined effect size for percentage weight change was −1.08% (95% CI: −1.66 to −0.49, I^2^ = 64.65%, *n* = 37), once adjusted for publication bias. Similarly, the combined effect size for percentage change in EE was −2.37%, (95% CI: −3.76 to −0.99, I^2^ = 87.77%, *n* = 46). Cognitive Behavioural Therapy showed the most promise for reducing weight and improving EE. Conclusions: Interventions to address EE showed promise in reducing EE and promoted a small amount of weight loss in adults living with overweight or obesity.

## 1. Introduction

In the UK, 33% of adults are overweight and 28% have obesity [1]. Overweight and obesity are defined as “abnormal or excessive fat accumulation that may impair health” [2]. In adults, Body Mass Index (BMI) is used as an estimate of body fat, with a BMI of 25 to 29.9 kg/m^2^ representing overweight, and a BMI of 30 kg/m^2^ and above being used to define obesity [3]. Living with obesity increases the risk of premature mortality, comorbidities, and reduces wellbeing [4]. Randomised Control Trials (RCTs) report that behavioural weight loss interventions targeting energy intake and physical activity produce modest weight losses of 7–10% in interventions lasting up to 30 weeks [5]. However, individuals may regain more than 80% of the weight lost within 5 years [6]. Behavioural Weight Loss (BWL) interventions are reported to be ineffective for individuals living with overweight/obesity [7]. These interventions do not address the psychological factors associated with eating [8] (such as emotional eating (EE) and/or binge eating behaviours) that are associated with overweight, obesity, and poor mental wellbeing [9].

There is no ubiquitous definition of EE, but EE is commonly defined as responding to negative feelings (e.g., stress, upset, or furiousness) for temporary comfort by overconsuming energy-dense and palatable foods [10,11,12,13]. Theories suggest such behaviours may become coping strategies and foreshadow unhealthy eating habits, causing weight gain [12,13]. Other theories suggest that reported positive emotions (e.g., joy or excitement) may also lead to EE and are commonly followed by negative feelings such as shame [14]. Macht (2008) proposed a five-way model of how emotions affect eating, including (1) emotional control of food choice, (2) emotional suppression of food intake, (3) impairment of cognitive eating controls, (4) eating to regulate emotions, and (5) emotionally congruent modulation of eating [15].

Whilst some individuals who engage in EE may meet the diagnostic criteria for Binge Eating Disorder (BED) [16] (eating in a discreet period of time, an amount of food that is definitively larger than what most individuals would eat in a similar period of time, under similar circumstances with a sense of lack of control over eating during the episodes) [17], others with EE episodes have no diagnosed eating disorder [14]. That said, EE is a form of disordered eating and is a developmental pathway to obesity [18]. Boredom, loneliness, anxiety, and stress are reported triggers to EE in females [19]. Reductions in EE are associated with greater weight loss in individuals living with obesity, especially in females [20]. At least 40% of individuals living with obesity are reported to experience EE [21]. Therefore, EE may be an important component for targeted weight loss interventions [22].

Regaining conscious control of eating is an important step in achieving a healthy body weight. Studies outline a range of psychological intervention approaches to address EE including Cognitive Behavioural Therapy (CBT), BWL, Mindfulness-Based Treatments (MBT), Acceptance-based Therapies [21,22,23,24], and combinations of the above.

A recent systematic review by Chew et al. (2022) synthesized weight loss interventions on EE and weight amongst adults with a Body Mass Index > 25 kg/m^2^ (BMI) [25]. The results indicated that CBT, BWL, and Mindfulness-based interventions were effective in improving EE and weight at one-year post-intervention. Pure Mindfulness approaches showed larger interventional effects than combination therapies [25]. The review also examined the effect of BWL interventions on binge eating, cognitive restraint/restrained eating, and BMI. The authors performed meta-regressions on all sufficient data, with the main outcome being weight loss. However, the authors limited their searches to RCTs, used a more limited search strategy, included participants with eating disorders, and excluded participants with pre-existing physical or mental health conditions.

The current review differs from existing work [25] by including interventions with components targeted at EE and broadening the search strategy to include all primary research designs, a wider range of search terms and settings, and no exclusion of participants with pre-existing physical or mental health conditions (other than eating disorders). Furthermore, our review converted outcome measures to percentage changes pre- and post-intervention, allowing a wider range of measurement tools to be included for EE.

The aims of this systematic review and meta-analysis were to understand:

1. Whether interventions that address EE are effective for achieving weight loss and/or improving EE in adults living with overweight or obesity.
2. Which psychological approach appears most effective for weight loss and/or improving EE for adults living with overweight or obesity.

## 2. Materials and Methods

The reporting of this review follows the Preferred Reporting Items for Systematic Reviews and Meta-Analysis (PRISMA) guidelines [26]. The PRISMA checklist [26] is included in Supplementary Table S1. The systematic review protocol is registered on the PROSPERO international prospective register of systematic reviews (CRD 42022302799).

### 2.1. Eligibility Criteria, Search Strategy and Selection of Studies

All studies that evaluated interventions with components that specifically addressed EE in adults with overweight or obesity were included. All primary research methodologies were included for consideration. Studies performing secondary data analysis on included RCTs were excluded. There were no date or geographical limits; however, searches were limited to papers written in English.

The inclusion criteria are reported in line with the PICOS guidance [27]. Participants: There were no limits on gender, ethnicity, or country of origin. However, studies had to be available in the English language. Studies were only included if >70% of participants did not meet criteria for BED or a clinically diagnosed eating disorders without EE as defined below. Similarly, >70% of the sample had to have a BMI of ≥25 kg/m^2^. Studies involving subjects under the age of 18 or animal studies were excluded. Interventions: Studies involving any psychological interventions with components specifically targeted at addressing EE were included. The definition of EE used was “the tendency to eat energy-dense and palatable foods, in response to negative emotions and/or eating to cope with unclear states of hunger and satiety with physiological changes and/or emotion regulation difficulties and/or various indicators of psychopathology including symptoms of anxiety and depression, negative self-concept, overeating”. Following discussion with the research team, studies involving medical interventions or medical devices were excluded. Furthermore, interventions that included components of EE post-bariatric surgery were excluded (pre-surgery were included). Pharmacological interventions and psychological therapies for weight loss that were not targeted at addressing EE were excluded, as were studies that more generally targeted food cravings. Comparison: Treatment as usual, waiting list control or no comparison group. Outcome: Studies reporting pre- and post-intervention data for weight and/or a measure of EE were included. Study design: Only studies with primary data were included. Systematic reviews, umbrella reviews, and scoping reviews were excluded [28], as were follow-up papers, protocol papers, non-peer reviewed studies and grey literature sources without scientific credibility (studies without robust designs or high risk of bias).

Keywords were generated by reviewing the literature in the field and consulting a leading Academic Psychologist in the field of Disordered Eating (Table 1). On the advice of the Academic Psychologist, a list of specific search terms was created for EE to ensure that appropriate citations were identified (Table 2).

**Figure 1:**
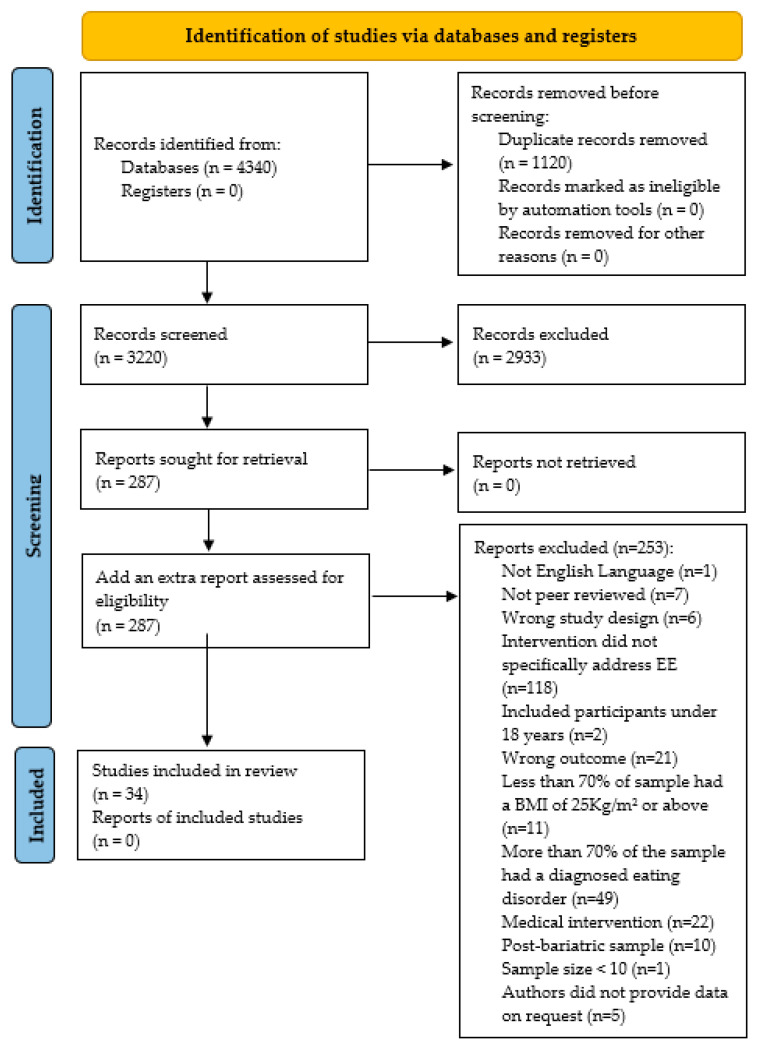
PRISMA flow diagram of search strategy for emotional eating interventions for adults living with overweight or obesity.

The following databases were searched: CINAHL, PsychInfo, MEDLINE, and EMBASE. Reference lists of the included papers were searched for any additional articles [28]. A grey literature search was also conducted of the first 100 hits on Google and MEDNAR (using “Emotional Eating in adults living with overweight or obesity” as the search term). All dates were searched. The searches were conducted from database inception to February 2022, ensuring that all eligible studies were included prior to data analysis. The PRISMA flow diagram [26] is shown in Figure 1. The searches were performed by X.Q.A. and uploaded into Rayyan, a web and app-based tool for systematic reviews [29]. Eligible studies were screened by two reviewers (X.Q.A. and J.S.). Both reviewers screened 100% of the titles and abstracts. In the second stage, all full-text articles were reviewed by X.Q.A., J.S., E.L.G., and G.T.T., with conflicts resolved through consensus. Approximately 10% of full-text articles required a consensus discussion.

**Table 2:** Summary of included studies.

Author, Date, Intervention Name and Country	Study Characteristics	Intervention to Address EE	Outcomes Reported
Afari et al. (2019) [30] MOVE + ACT USA	Design: RCT Sample size: *n* = 88 (85 completers) Mean age: 57.3 years (SD: 9.9). Gender: 76.1% male, Ethnicity: 70.5% Caucasian, 17% African American, and 12% Hispanic	Interventions: Acceptance and Commitment Therapy (ACT) Behavioural Weight Loss (BWL) Both length of interventions: 8 weeks in person, 90 days by telephone	Weight and EE
Annesi, et al. (2016) [31] LEARN USA	Design: Individually randomized group treatment trial Sample size: *n* = 103 Mean age: 47.8 years (SD: 7.9) Gender: 100% female Ethnicity: 84% White, 12% African American, and 4% other	Interventions: Mindfulness (personal contact group vs. self-help). Length of the intervention: 6 months, in person	Weight and EE
Annesi (2019) [32] USA	Design: Individually randomized group treatment trial Sample size: *n* = 152 Mean age: 48.6 years (SD:7.0) Gender: 100% female Ethnicity: 80% White, 15% Black, 5% other	Interventions: Group 1: Behavioural Weight Loss (BWL) Group 2: Behavioural Weight Loss (BWL) + Cognitive Behavioural Treatment (CBT) Group 3: Behavioural Weight Loss (BWL) + Cognitive Behavioural Treatment (CBT). Length of interventions: Group 1: 28 weeks, phone calls Group 2: 58 weeks, in person Group 3: 99 weeks, phone calls and in person	EE only
Bacon et al. (2005) [33] Health at Every Size USA	Design: Individually randomized group treatment trial Sample size: *n* = 35 completers at post-intervention. Mean age: HAES: 40.4 (SD:4.4); Diet: 41.4 years (SD:3.0) Gender: 100% female Ethnicity: Not reported	Interventions: Diet Group (BWL) Health at Every Size (HAES) Group (Acceptance-based) Length of intervention: 6 months, group	Weight and EE
Carbine et al. (2021) [34] USA	Design: RCT Sample size: *n* = 100 Mean age: 28.05 years (SD:7.56) Gender: 53% Female Ethnicity: 81% Caucasian, 14% Hispanic, 5% other	Interventions: Food Specific ICT (i.e., inhibiting responses to high-calorie foods) (Other therapy) Generic ICT (i.e., inhibiting responses to everyday items) (Other therapy) Length of intervention: 4 weeks, in person	Weight and EE
Carpenter et al. (2019) [35] Mind Your Weight USA	Design: Individually randomized group treatment trial, pilot study Sample size: *n* = 75 Mean age: 47.3 years (SD:10.0) Gender: 92% Female Ethnicity: 65.3% White, 26.7% Black, 6.7% Hispanic, 1.3% Asian	Interventions: Mindfulness weight loss program (Mind Your Weight) (Mindfulness) Behavioural weight loss program (Weight Talk^TM^) (BWL) Length of intervention: 6 months, telephone-based counselling	EE only
Chung et al. (2016) [36] USA	Design: Single group design, longitudinal study Sample size: *n* = 22 Mean age: 50.14 years (SD:9.0) Gender: 100% Female Ethnicity: 100% African American	Intervention: Mindful Eating Length of intervention: 12 weeks, group and in person	Weight and EE
Daubenmier et al. (2016) [37] SHINE USA	Design: RCT Sample size: *n* = 194 Mean age: 47.5 years (SD:12.7) Gender: 80% Female Ethnicity: 59.3% European, 12.9% African, 9.8% Asian/Pacific Islander, 11.9% Latina/Latino, 1% Native American, 5.1% other	Intervention: Mindfulness-based weight loss intervention Behavioural Weight Loss as active control group Length of intervention: Both interventions included 16 sessions lasting 2 to 2.5 h (12 weekly, 3 biweekly, and 1 monthly) and one all-day session (6.5 and 5 h in the Mindfulness and control interventions, respectively) over 5.5 months.	Weight only
Forman et al. (2013) [38] Mind your Health USA	Design: RCT Sample size: *n* = 128 (99 completers post-intervention) Mean age: 45.69 years (SD:12.81) Gender: Not reported Ethnicity: 62.3% Caucasian, 24.6% African American, 1.6% Asian, 3.8% Hispanic	Interventions: Acceptance-based Behavioural Treatment (Acceptance-based) Standard Behavioural Treatment (Behavioural therapy) Length of intervention: 40 weeks, group and in person	EE only
Frayn et al. (2020) [23] Switzerland	Design: Single group design Sample size: *n* = 32 Mean age: 46.71 years (SD:13.43) Gender: 87.5% Female, 12.5% Male Ethnicity: 78.1% Caucasian, 3.1% Middle Eastern, 3.1% Black, 3.1% Hispanic, 12.5% other	Intervention: Acceptance and Commitment Therapy (Acceptance-based) Length of intervention: 1 day, group and in person	EE only
Goldbacher et al. (2016) [24] USA	Design: Individually randomized group treatment trial Sample size: *n* = 79 Mean age: 45.6 years (SD:10.5) Gender: 95% Female, 5% Male Ethnicity: 80% African American, 11% White, 4% Hispanic, 5% other	Interventions: Behavioural Weight Loss Treatment (BWL) Enhanced Behavioural Treatment (Behavioural Therapy) Length of intervention: 20 weeks, group and in person	Weight and EE
Hanson et al. (2019) [39] UK	Design: Single group design Sample size: *n* = 53 (33 completers) Mean age: 44.4 years (SD:11.0) Gender: 78.8% Female Ethnicity: Not reported Mean weight: 126.3 kg (SD:36.1)	Intervention: Mindfulness Compared to control group Length of intervention: 8 weeks, group and in person	Weight and EE
Hawkins et al. (2021) [40] POWER-UP USA	Design: Single group design Sample size: *n* = 48 Mean age: 43.58 years (SD:1.50) Gender: 85.4% Female Ethnicity: Not reported	Intervention: Acceptance-based Behavioural Therapy (Acceptance-based) Length of intervention: 23 weeks, group and in person	Weight and EE
Hepdurgun et al. (2020) [41] Turkey	Design: Individually randomized group treatment trial Sample size: *n* = 51 in the intervention group Mean age: 40.1 years (SD:9.96) Gender: 80.4% Female, 19.6% Male Ethnicity: Not reported	Intervention: Internet-based Behavioural Therapy Compared to routine care (healthy eating and physical activity information) by email Length of intervention: 8 weeks, online and in person	Weight only
Hunot-Alexander et al. (2021) [42] UK	Design: Single group design with qualitative evaluation Sample size: *n* = 37 (weight data reported for 32) Mean age: 48.3 years (SD:10.9) Gender: 93.8% Female, 6.3% Male Ethnicity: 90.6% White, 9.4% non-White	Intervention: Appetitive Trait Tailored Intervention (Other therapy) Length of intervention: 8 weeks, in person	Weight only
Kearney et al. (2012) [43] USA	Design: Single group design Sample size: *n* = 48 (38 completers post-intervention) Mean age: 49 years (SD:10.7) Gender: 87.5% Male Ethnicity: 85.4% White, 4.2% Black, 6.3% Hispanic, 4.2% Asian/Pacific-Islander/Native American	Intervention: Mindfulness-based stress reduction (Mindfulness). Length of intervention: 2 months, group and in person	Weight and EE
Keränen et al. (2009) [44] LITE Finland	Design: Individually randomized group treatment trial Sample size: *n* = 20 completers in the intervention group Mean age: 52 years (SD: 7.0) Gender: 25% Male Ethnicity: Not reported	Intervention: Intensive Counselling (with components of EE) Compared to Short-term Counselling (no components of EE) Length of intervention: 20 weeks, group and in person	Weight only
Kidd et al. (2013) [45] USA	Design: Single group design Sample size: *n* = 12 Mean age: 51.8 years (SD: 9.1)p Gender: 100% Female Ethnicity: 58.3% African American, 41.7% White	Intervention: Mindful Eating Length of intervention: 8 weeks, in person	Weight and EE
Kim et al. (2021) [46] Healthy Life Plan Korea	Design: RCT Sample size: *n* = 583 (369 completers post-intervention) Mean age: 53.68 years (SD:10.12) for the IG and 53.94 years (SD: 10.18) for the MG. Gender: 61.6% Female, 38.4% Male Ethnicity: Not reported	Intervention: Intensive intervention group (IG). Received a multi-component intervention to reduce abdominal obesity by mainly focusing on dietary attitude and dietary behaviour change and a minimal information intervention (Other therapy) Minimal information intervention group Received a brief explanation of health status and a simple recommendation for a lifestyle change (Other therapy) Length of intervention: 6 months, group and in person	EE only
Lillis et al. (2016) [47] USA	Design: RCT Sample size: *n* = 162 Mean age: 50.2 years (SD: 10.9) Gender: 85% Female, 15% Male Ethnicity: 5% Black/African American, 6% Hispanic, 1% Asian, 88% Caucasian	Intervention: Standard Behavioural Treatment (Behavioural Therapy) Acceptance-based Behavioural Intervention (ABBI Acceptance-based) Length of intervention: 24 months, group and in person	Weight only and EE
Malkina-Pykh (2012) [48] Russia	Design: RCT Sample size: *n* = 104 (58 completers at post-intervention) Mean age: 37.6 years (SD:6.7) Gender: 69% Female, 31% Male Ethnicity: Not reported	Intervention: Cognitive Behavioural Therapy (CBT) +/− Rhythmic Movement Therapy (RMT). This was added for half of participants who showed no improvement with CBT after 6 months (*n* = 30). The remaining 28 participants who did not respond to CBT after 6 months continued with CBT. Length of intervention: 24 bi-weekly sessions (48 weeks), in person	EE only
Manzoni et al. (2009) [49] Italy	Design: RCT Sample size: *n* = 40 in the two intervention groups Mean age: Not reported Gender: 100% Female Ethnicity: Not reported	Intervention: Relaxation training—traditional (imagination condition) Relaxation training (virtual reality condition) Compared to standard hospital-based care Length of intervention: 5 weeks, in person during an inpatient stay	Weight only
Mason et al. (2018) [50] USA	Design: Single group design Sample size: *n* = 104 (61 completers at post-intervention) Mean age: 46.07 years (SD:14.64) Gender: Not reported Ethnicity: 68.3% White, 4.8% Black, 10.6% Hispanic/Latino, 9.6% Asian/Pacific Islander, 0.0% Native American, 1.0% declined to answer	Interventions: Mindfulness Length of intervention: 28 days, via a mobile phone	Weight and EE
Moraes et al. (2021) [51] Brazil	Design: RCT Sample size: *n* = 64 in the two intervention groups of interest Mean age: EH: 35.98 years (SD:6.76); IT + CBT: 36.18 years (SD:2.75) Gender: EH: 81.8% Female, 18.2% Male; IT +CBT: 77.4% Female, 22.6% Male Ethnicity: Not reported	Interventions: Education and Health Group (EH) (BWL) Interdisciplinary Therapy plus Cognitive Behavioural Therapy (IT + CBT) Compared to a physical activity program (no elements of EE, control) Length of intervention: 30 weeks, group (EH), group and individual (IT + CBT)	Weight and EE
Niemeier et al. (2012) [52] USA	Design: Single group design pilot Sample size: *n* = 21 (18 completers at post-intervention) Mean age: 52.2 years (SD:7.6) Gender: 90.5% Female, 9.5% Male Ethnicity: 90% non-Hispanic, 4.8% Hispanic, 4.8% other	Intervention: Acceptance-Based Behavioural Intervention (ABBI BWL + Acceptance-based) Length of intervention: 24 weeks, group	Weight and EE
Paans et al. (2020) [53] MooDFOOD Netherland	Design: RCT Sample size: *n* = 372 for intervention group Mean age: 47.8 years (SD:12.6) Gender: 78.2% Female Ethnicity: Not reported	Intervention: Food-related Behavioural Activation Therapy (Behavioural approach) Groups: Multi-nutrient supplement + FBA Placebo supplement + FBA Multi-nutrient supplement Placebo supplement Length of intervention: 1 year, 15 individual sessions, 6 group sessions	Weight and EE
Palmeira et al. (2017) [54] Kg-free Portugal	Design: RCT Sample size: *n* = 27 for intervention group Mean age: 41.97 years (SD: 8.79). Gender: 100% women Ethnicity: Not reported	Intervention: Acceptance and Commitment Therapy (Acceptance-based) Compared to treatment as usual Length of intervention: 12 weeks, group and in person	EE only
Rieger et al. (2017) [55] Australia	Design: RCT Sample size: *n* = 201 (118 completers post-intervention) Mean age: 47.01 years (SD:11.52) Gender: 73.6% Female	Intervention: Cognitive Behaviour Therapy (CBT): Weight loss alone (CBT) Weight loss with the addition of a Support Person (CBT). Length of intervention: 12 months, group and in person	Weight and EE
Roosen et al. (2012) [21] Netherlands	Design: Pilot, single group design Sample size: *n* = 35 Mean age: 39.2 years (SD:11.02) Gender: 86% Female, 15% Male Ethnicity: Not reported	Intervention: Dialectical Behaviour Therapy (DBT) Length of intervention: 20 weeks, group and in person	EE only
Salvo et al. (2021) [56] MB-EAT Brazil	Design: Pilot, single group design, with mixed-methods evaluation Sample size: *n* = 20 Mean age: 48.15 years (SD:8.57) Gender: 100% Female Ethnicity: Not reported	Intervention: Mindfulness-based Eating Awareness Training (Mindfulness) Length of intervention: 13 weeks, group	Weight only and EE
Sampaio et al. (2021) [57] Brazil	Design: RCT Sample size: *n* = 27 for intervention group Mean age: 49 years (SD:11.0) Gender: 100% Female Ethnicity: 7.4% White, 40.7% Black, 51.9% mixed	Intervention: Meditation practice. It presumes that health is related to balance and integration of the physiological, emotional, cognitive, behavioural, and spiritual aspects of human functioning (Mindfulness) Compared to a control group Length of intervention: 7 months, group	EE only
Spadaro et al. (2017) [58] USA	Design: RCT Sample size: *n* = 46 Mean age: 45.2 years (SD:8.2) Gender: 87% Female, 13% Male Ethnicity: 78.3% Caucasian, 21.7% African American	Intervention: Behavioural Weight Loss Programs (BWL) Behavioural Weight Loss Program + Mindfulness Meditation (BWL + Mindfulness) Length of intervention: 6 months, group	Weight and EE
Tham and Chong (2020) [59] Medical & Mind Weight Loss Redefine CBT Programme Australia	Design: Single group design Sample size: *n* = 120 Mean age: Not reported Gender: 57.5% Female, 42.5% Male	Intervention: Cognitive Behavioural Therapy (CBT) Length of intervention: 26 weeks, online	Weight and EE
Thomas et al. (2019) [60] POWER and MORE POWER USA	Design: RCT Sample size: *n* = 51 Mean age: 57.92 years (SD: 10.04) Gender: 100% Female Ethnicity: 96% White, 2% Black/African American, 2% Hispanic/Latino	Intervention: Exercise and nutrition counselling POWER (BWL) Exercise and nutrition counselling + MOREPOWER (Mindfulness) Length of intervention: 10 weeks, group and in person	Weight and EE

### 2.2. Data Extraction

Data were extracted from eligible studies and recorded in an Excel spreadsheet to obtain relevant data regarding: the aim of the study; study population characteristics; study design; sample population; intervention approach; duration of intervention; main findings; author conclusions; and author contact details. Two reviewers (X.Q.A. and J.S.) independently extracted the data for completeness and accuracy, and any conflicts were resolved through consensus. A third reviewer (G.T.T.) checked the final data extraction.

### 2.3. Critical Appraisal

Two reviewers (X.Q.A., and E.L.G.) independently assessed the study quality using the CASP Critical Appraisal Tools [61] for RCTs and JBI Checklist for Quasi-Experimental Studies [62] for single group design studies. Studies meeting at least 75% of the appraisal criteria were considered to be of high methodological quality; those meeting 50–74.9% were considered medium quality; and those meeting less than 50% were considered low quality.

### 2.4. Synthesis of Results and Analytical Strategy

The quantitative data were reviewed and were deemed sufficient for meta-analysis of weight outcomes and EE outcomes. They were converted into percentage change due to the variation in tools to measure EE and the wide variation in baseline data. The primary outcome of this review was mean weight change pre- and post-intervention, as opposed to BMI which has a high specificity but low sensitivity for assessing obesity [63]. The secondary outcome was change in mean EE score pre- and post-intervention.

Where data were missing, the authors were emailed to request this data [21,23,32,38,42,46,48,54,57,64]. The Meta-Essentials Workbooks for Meta-analysis version 1.5 were used for the meta-analysis [65]. The results of individual studies are outlined in a table of frequencies in Supplementary Table S2.

## 3. Results

After removal of duplicates, 3220 citations were identified. Following the title and abstract screen, 287 full text papers were evaluated against the eligibility criteria and 253 papers were excluded. The reasons for exclusion are summarized in Figure 1, and fully reported in the Hierarchy of Exclusion in Supplementary Table S3. After screening the grey literature, no further scientifically credible studies were identified, therefore the PRISMA diagram [26] (Figure 1) includes databases and registers only.

### 3.1. Study and Participant Characteristics

#### 3.1.1. Study Characteristics

Thirty-four studies were included in this review from database inception up to February 2022 [21,23,24,30,31,32,33,34,35,36,37,38,39,40,41,42,43,44,45,46,47,48,49,50,51,52,53,54,55,56,57,58,59,60] Fifty-nine percent were published from 2017 onwards (*n* = 20) [23,30,32,34,35,39,40,41,42,46,50,51,53,54,55,56,57,58,59,60] and 21% were published from 2021 onwards (*n* = 7) [34,40,42,46,51,56,57]. The remaining studies (41%) were published before 2017 [21,24,31,33,36,37,38,43,44,45,47,48,49,52]. Fifty-three percent were published in the USA (*n* = 18) [24,30,31,32,33,34,35,36,37,38,40,43,45,47,50,52,58,60] with the remainder being published in Brazil (*n* = 3) [51,56,57]; Australia (*n* = 2) [55,59]; the Netherlands (*n* = 2) [21,53]; the United Kingdom (*n* = 2) [39,42]; Finland (*n* = 1) [44]; Italy (*n* = 1) [49]; Korea (*n* = 1) [46]; Portugal (*n* = 1) [54]; Russia (*n* = 1) [48]; Switzerland (*n* = 1) [23]; and Turkey (*n* = 1) [41]. Forty-three percent were RCTs (*n* = 15) [30,34,37,38,46,47,48,49,51,53,54,55,57,58,60]; 34% were single group designs (*n* = 12) [21,23,36,39,40,42,43,45,50,52,56,59]; and 20% were individually randomised group treatment trials comparing two or more EE interventions (*n* = 7) [24,31,32,33,35,41,44].

#### 3.1.2. Participant Characteristics

A total of 3229 participants were included in this review, with an age range from 18 to 76 years. Sixty-six percent of participants were female (*n* = 2121); 27% were male (*n* = 876); and gender was not reported for 7% of participants (*n* = 232). Ethnicities were 32% White (*n* = 1021); 8% Black (*n* = 256); 3% Hispanic/Latino (*n* = 89); 1% Asian and Pacific Islander (*n* = 36); 0.5% mixed (*n* = 14); and 1.5% other (*n* = 48). Ethnicity was not reported for 54% of participants (*n* = 1765).

### 3.2. Variables and Measurement Tools

#### 3.2.1. Weight and BMI

Twenty-five studies reported pre- and post-intervention means for weight. Most studies included participants with a BMI over 25 kg/m^2^, with no upper BMI limit for inclusion. However, the study by Lillis et al. (2016) excluded participants with a BMI over 50 kg/m^2^ [47].

#### 3.2.2. Emotional Eating

Thirty studies reported pre- and post-intervention data for EE scores. A wide variety of tools were used to measure EE (*n* = 11). Data were extracted from the EE subscale of each tool (where provided). The most commonly used tool was the Dutch Eating Behaviour Questionnaire (DEBQ) [66] (*n* = 9) [21,23,30,34,46,48,51,57,60]. This was followed by the Emotional Eating Scale (EES) [67] (*n* = 5) [24,31,32,38,40]; the Three-Factor Eating Questionnaire (TFEQ) [68] (*n* = 5) [43,44,50,53,54]; the Eating Inventory [69] (*n* = 4) [33,47,52,58]; the Mindful Eating Questionnaire (MEQ) [70] (*n* = 3) [35,36,45]; the Binge Eating Scale (BES) [71] (*n* = 1) [55]; the Emotional Eater Questionnaire [72] (*n* = 1) [59]; the Eating Attitudes Test (EAT-26) [73] (*n* = 1) [56]; the Emotional Overeating Questionnaire (EOQ) [74] (*n* = 1) [49]; the Whole Person Integrated Eating Questionnaire (WPIEQ) [75] (*n* = 1) [39]; and the Food Craving Questionnaire—Trait-Reduced (FCQ-T-R) [76] (*n* = 1) [50]. Three studies did not report outcomes for EE, and only reported on weight outcomes [37,41,42].

### 3.3. Interventions

Several studies examined more than one psychological intervention. The most utilised psychological interventions were Behavioural Weight Loss (BWL) and Mindfulness. Table 2 provides full details of the interventions used in each study. The main intervention categories are outlined below.

#### 3.3.1. Behavioural Weight Loss

Ten studies used BWL [24,30,32,33,35,37,44,51,58,60]. BWL interventions include specific behavioural strategies (e.g., portion control and regular meals) to modify eating behaviour [77].

#### 3.3.2. Mindfulness

Mindfulness was used in 10 studies [31,35,36,37,39,43,45,50,56,57]. Mindfulness interventions in this review included general Mindfulness techniques drawn from Mindfulness-based stress reduction, along with specific mindful eating training derived from Mindfulness-Based Eating Awareness Training [78]. The Mindful Eating techniques involved identifying and responding adaptively to food cravings, and providing skills for emotion regulation that would allow individuals to sit with, rather than trigger EE [79].

#### 3.3.3. Acceptance-Based Interventions

Seven studies involved Acceptance-based interventions [23,30,33,38,40,47,54]. Acceptance-based interventions build upon the behavioural skills used in BWL programs by adding components derived from ACT. The three principal components of Acceptance-based interventions are distress tolerance, mindfulness, and commitment enhancement [80].

#### 3.3.4. Combined Interventions

Six studies used combined therapies [32,48,51,52,58,60], for example, a combination of Cognitive Behavioural Therapy (CBT) and BWL.

#### 3.3.5. Behavioural Therapy

Five studies used Behavioural Therapies [24,38,41,47,53] such as Standard Behavioural Therapy (SBT) (e.g., counselling on diet and physical activity), a BWL intervention generally focus on increasing awareness of one’s eating behaviour through dietary self-monitoring and goal setting [5]. This category also included Enhanced Behavioural Treatment (EBT) which integrates SBT components with techniques that specifically target EE [24].

#### 3.3.6. Cognitive Behavioural Therapy

Three studies involved CBT [48,55,59]. The European Society of Physical and Rehabilitation Medicine [81] guidelines strongly recommend CBT as the gold standard psychological intervention for obesity.

#### 3.3.7. Dialectical Behavioural Therapy

One study used Dialectical Behavioural Therapy (DBT) [21]. The principal components for DBT are mindfulness, emotion regulation, and distress tolerance, which required coaching with a professional [82].

#### 3.3.8. Other Therapies

Five studies used other therapies such as Appetitive Trait Tailored Intervention [34,37,42,46,49].

### 3.4. Meta-Analysis

#### 3.4.1. Weight

**Figure 2:**
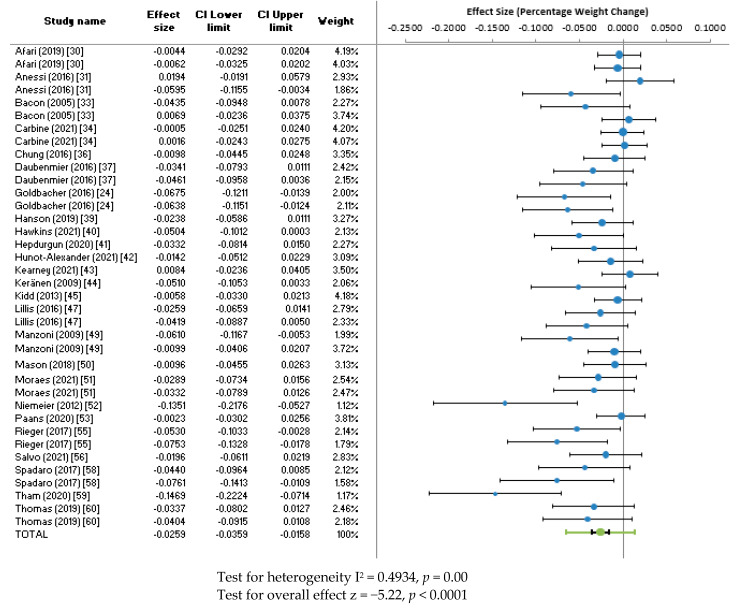
Forest plot for percentage weight change.

Twenty-five studies reported pre- and post-intervention mean weight or provided this data upon request. Twenty-five studies, reporting on 37 different interventions were therefore included in the weight meta-analysis (some studies reported on multiple interventions). The combined effect size for percentage weight change across all interventions was −2.59% (95% CI: −3.59 to −1.58, z = −5.22, *p* < 0.0001, *n* = 37). The heterogeneity of the studies was moderate (I^2^ = 49.34%). A forest plot for the combined effect size of all interventions is shown in Figure 2.

A sub analysis of effect size by intervention category showed considerable variation in the combined effect size for weight, with CBT showing the most promise (−9%, 95% CI: −20 to 3, *n* = 3), followed by combined interventions (−6%, 95% CI: −13 to 1, *n* = 4); BWL (−3%, 95% CI: −5 to −1, *n* = 8); Behavioural Therapies (−3%, 95% CI: −7 to 1, *n* = 4); Acceptance-based interventions (−2%, 95% CI: −6 to 3, *n* = 4); and Mindfulness (−1%, 95% CI: −3 to 0, *n* = 9).

**Figure 3:**
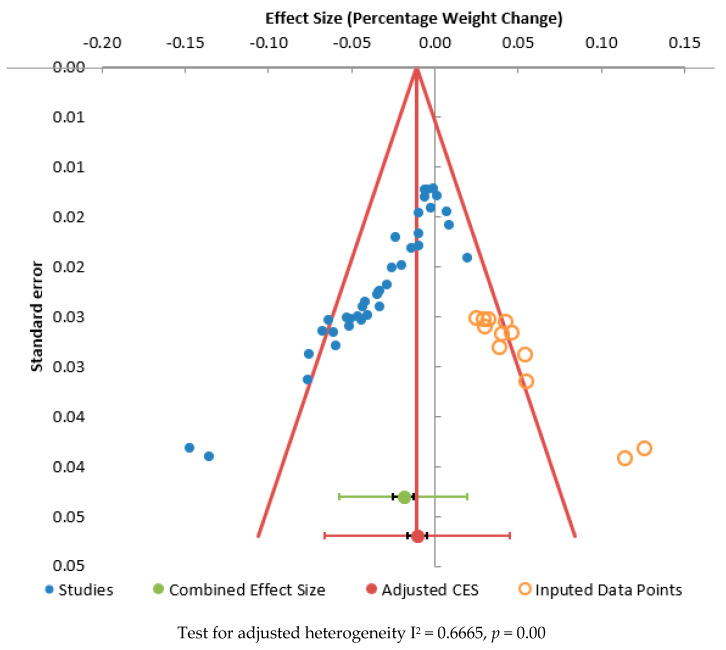
Funnel plot for effect size against standard error for percentage weight change.

Figure 3 shows a funnel plot of the combined effect size for weight against standard error. The Egger Regression test was significant for publication bias (*p* = 0.000). This suggests there was significant publication bias within this meta-analysis. Applying the Trim and Fill [83] procedure to estimate the unbiased effect size, the adjusted effect size was −1.08% (95% CI: −1.66 to −0.49, I^2^ = 64.65%, *n* = 37).

#### 3.4.2. Emotional Eating Score

**Figure 4:**
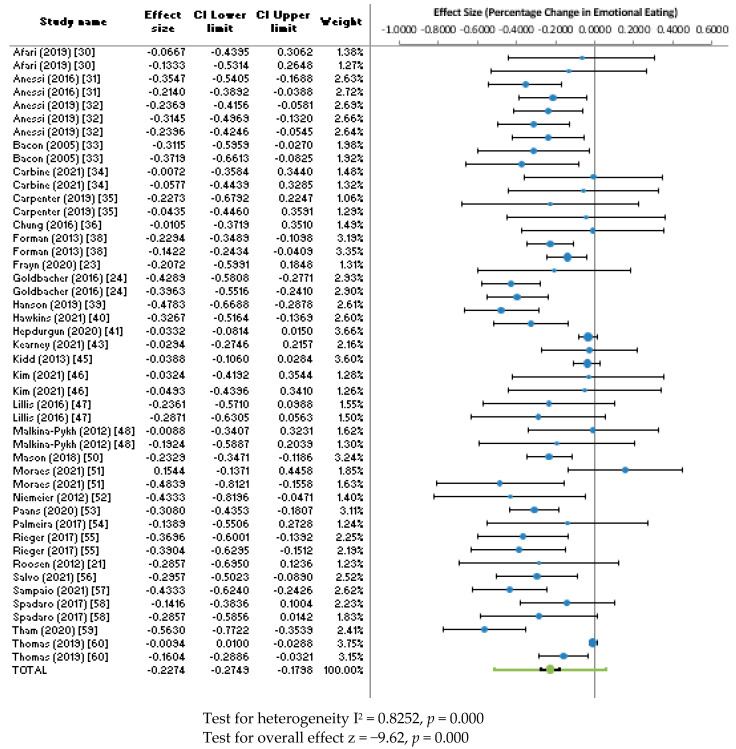
Forest plot for effect size of percentage change in emotional eating.

Thirty studies reported pre- and post-intervention mean EE scores or provided this data upon request. Thirty studies, reporting on 46 different interventions were included in the meta-analysis (some studies reported on multiple interventions). The combined effect size for percentage change in EE scores across all interventions was −22.74% (95% CI: −27.49 to −17.98, z = −9.62, *p* < 0.0001, *n* = 46). The heterogeneity of the studies was considerable (I^2^ = 82.52%). A forest plot for the combined effect size of all interventions is shown in Figure 4.

A sub analysis of the effect size by intervention category showed considerable variation in the combined effect size for the EE score, with CBT interventions showing the most promise (−38%, 95% CI: −63 to −13, *n* = 5), followed by Acceptance-based interventions (−25%, 95% CI: −32 to −18, *n* = 7); Mindfulness (−24%, 95% CI: −36 to −12, *n* = 10); combined interventions (−23%, 95% CI: −32 to −14, *n* = 6); Behavioural Therapies (−21%, 95% CI: −40 to −3, *n* = 5); and BWL (−15%, 95% CI: −31 to 0, *n* = 8). There was only one study that reported on DBT and therefore, a combined effect size was not calculated for this intervention.

**Figure 5:**
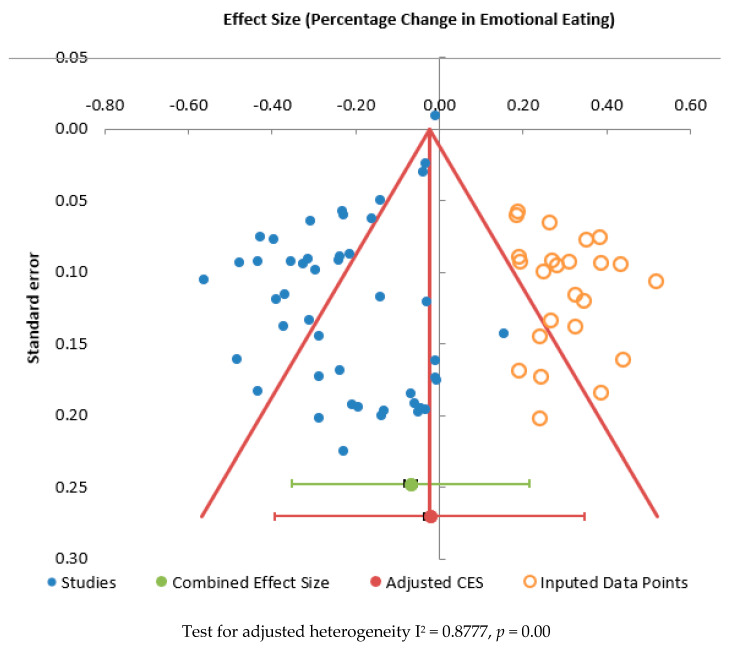
Funnel plot for effect size against standard error for percentage change in emotional eating score.

Figure 5 shows a funnel plot of the combined effect size for EE against standard error. Considerable heterogeneity can be seen by the points outside of the lines. The Egger Regression test was significant for publication bias (*p* = 0.000). This suggests there was significant publication bias within this meta-analysis. Applying the Trim and Fill [83] procedure to estimate the unbiased effect size, the adjusted effect size was −2.37%, (95% CI: −3.76 to −0.99, I^2^ = 87.77%, *n* = 46).

### 3.5. Methodological Quality

All 22 RCTs or individually randomized group treatment trials were considered high quality, meeting at least 75% of the CASP criteria, with 9 studies meeting 100% of the criteria [24,30,34,35,37,44,46,47,57]. For non-randomised intervention studies, 7 studies were considered high quality, meeting at least 75% of the criteria [23,36,39,40,43,56,59]; 4 studies were medium quality, meeting 50–74.9% of the criteria [21,45,50,52]; and 1 study was low quality, meeting less than 50% of the criteria [42]. The full assessment of the critical appraisal results is reported in Supplementary Tables S4 and S5.

## 4. Discussion

This systematic review and meta-analysis aimed to investigate whether interventions to address EE are effective for achieving weight loss and/or reducing EE in adults living with overweight or obesity. In summary, 34 studies were included in this review, with half being published in the USA. The combined effect size for all interventions on percentage weight change was −1.08%, once adjusted for publication bias. The combined effect size for percentage change in EE was −2.37%, once adjusted for publication bias. CBT showed the most promise for weight loss and improving EE in the sub analyses. Both effects sizes were small, which was expected due to the short-term nature of the interventions included in the studies. Furthermore, the authors anticipated a larger effect size for the change in EE, as a change in eating behaviour would be an expected precursor to longer term weight loss.

There was significant variability in the 34 included studies in terms of research design, methodology, outcome measures, measurement tools, and intervention delivery/fidelity. This review concurred with Chew et al. (2022) [25] in that there is inconsistency in use of standard protocols for psychotherapeutic interventions. Our review identified a wide variety of self-reported tools have been developed and validated to measure EE (*n* = 11), with the Dutch Eating Behaviour Questionnaire (DEBQ) [66] being the most commonly used tool. Furthermore, there was wide variation in the length of intervention (1 day to 24 months); the setting (in person, groups, telephone, internet, self-help, and inpatient admissions); amount of support the participants received (self-help to 1:1 weekly support); and the amount of focus on EE within the intervention. Future research therefore needs to explore some of these factors and better understand the active behaviour change components of effective interventions.

The searches in this review identified 3220 records (after duplicates were removed), which is higher than the review by Chew et al. (2022) (*n* = 528) [25]. More studies were included in our meta-analysis (*n* = 25 for weight, and 30 for EE score) compared to Chew et al. (*n* = 19). This is likely due to the broader search strategy as described in Section 1.

### 4.1. Intervention Effects on Weight

Although health education is important, the psychological triggers and context of eating need to be considered in obesity treatment [51]. Several psychological interventions such as Mindfulness, ACT, CBT, and DBT have shown promising results in reducing EE and facilitating weight loss [84]. Our meta-analysis reported a small effect on weight (−1.08% from pre- to post-intervention). However, a meta-analysis by Chew et al. (2022) [25] reported no significant effect of EE interventions on weight outcomes (*p* = 0.12). Our review found that CBT showed the most promise for weight loss (−9%), followed by combined interventions (−6%). Previous studies without a focus on EE have largely found that CBT is effective for weight loss [80,85]. The average length of CBT interventions in this review lasted between six weeks and six months, which is longer than some of the other interventions used. This may explain why CBT (and combined interventions that include elements of CBT) showed more promise for weight loss compared to other interventions. Chew et al. [25] reported that purely Mindfulness-based interventions showed a higher interventional effect size for weight compared to other interventions. This differs from the findings of our review where Mindfulness-based interventions showed the least promise for percentage weight change. Furthermore, Chew et al.’s review found that combined interventions had counter-productive effects on weight whereas our review found they showed a degree of promise. This may be due to the methodological differences between the reviews, as Chew used Hedges g to assess the effect size (kg) pre-and post-intervention, whereas our review converted weight changes into percentages to enable us to include more studies and aid interpretation of results (there was a wide variation in mean baseline weights reported between the included studies).

### 4.2. Interventions for Improving Emotional Eating

The meta-analysis showed a higher percentage change in EE than weight (−2.37%) following intervention. This is encouraging as a reduction in EE is an anticipated precursor to weight loss and improved mental wellbeing. CBT showed the most promise in reducing EE (−38%), followed by Acceptance-based interventions (−25%). A possible intervention approach for adults living with obesity and EE is to educate individuals to understand and recognise EE, instead of restricting dietary intake which often exacerbates the issue [84]. Both CBT and Acceptance-based interventions may reduce EE episodes by recognizing stressors and resultant emotions and replacing the urge to eat with alternative positive actions [38,86]. CBT is a common approach to support change in EE by keeping a food and mood diary to identify the triggers and situational context of eating [87,88]. Glisenti and Strodl [89] suggested that DBT may be more effective than CBT in reducing EE as DBT focusses on both emotion regulation and Mindfulness [89], but there was only one study identified in this review that used DBT. Forman et al. [90] suggested Acceptance-based strategies may eliminate and reduce EE compared to BWL interventions. One study indicated a 1-day ACT workshop can be beneficial in reducing EE, however acceptance and value clarification skills may require more time to incarnate and develop as a longer term intervention [23]. Numerous studies concluded that changing eating behaviour may lead to improvements in both physical and mental health outcomes [91,92,93]. Mindfulness was the most common intervention used by authors. This has been shown in existing research to reduce EE by regulation of emotions [94,95,96]. Studies have shown mixed results for Mindfulness on eating behaviour [40,97]. When considering only Mindfulness-based interventions, individuals seeking to lose weight may benefit more from Mindfulness skills emphasizing increased awareness, acceptance, and overriding of hedonic drives to eat, rather than those promoting reliance on homeostatic cues to reduce consumption [71]. One study in this review [34] examined Mindfulness plus/minus professional contact and resulted a positive improvement in EE; however, the authors reported an increase in weight in participants without professional contact. Chew et al. (2022) [25] reported a small to medium interventional effect for Mindfulness interventions on EE post-intervention (*p* = 0.01). They also reported that pure Mindfulness interventions showed a higher interventional effect size for EE, when compared to other interventions or combinations of interventions. Mindfulness is a key component in Acceptance-based interventions and DBT [94]. Studies reported combined interventions such as CBT and Mindfulness may be advantageous as they enable individuals to explore behaviour change techniques whilst also learning acceptance strategies to reduce EE [24,95]. Greater awareness of emotions and stress enabled individuals to improve regulation of eating and make healthier choices, which leads to fewer EE episodes [45].

### 4.3. Intervention Setting and Mode of Delivery

The majority of participant contact in the studies was face-to-face, in a group setting, and was delivered by trained professionals. Comparator groups included personal contact with trained professionals, and manual-based treatment with telephone support. Three studies compared personal contact to remote delivery/self-help interventions [31,46,55]. However, there were no consistent findings between the modes of delivery. Further research is therefore required to determine the impact of the setting and mode of delivery on weight and EE in adults with overweight or obesity. Existing evidence from studies reported that personal contact with a health professional increases weight loss success [96,97] and motivation [98]. However, online CBT studies do report that remote delivery was useful for people living in certain locations or those who were unable to attend regularly face-to-face sessions [99,100]. Considering the recent COVID-19 pandemic, this online platform is likely to become more popular with service users [101] and therefore all methods of delivery should be considered in clinical practice. Whilst CBT showed the most promise for reducing weight and improving EE, it is important to acknowledge that implementing CBT in treatment would require longer intervention times and requires specialist healthcare professionals with extensive training. It is therefore vital that further work is undertaken to explore practical ways in which healthcare professionals supporting adults with overweight or obesity might implement CBT approaches to address EE. One example would be through brief, manualised, or online self-help approaches (guided self-help).

Six of the interventions in this review were multi-component and therefore future research should aim to identify the active components that are most effective for improving EE and achieving weight loss. An important first step in weight management practice would be the development of an agreed pathway for EE.

### 4.4. Screening Tools for Emotional Eating

This review identified a wide variety in the available tools to measure EE (*n* = 11). The most commonly used tool was the Dutch Eating Behaviour Questionnaire (DEBQ) [66]. Different validated tools may allow for the comparison of the predictive validity of EE in relation to weight outcomes to see which construct has a greater effect on weight loss [84]. However, the use of different tools in the included studies highlights the need for some consensus amongst health professionals and researchers regarding the most appropriate tool to use in weight management practice [102]. Further research is therefore required to scope the available tools; their reliability and validity; their strengths and weaknesses; and their feasibility and acceptability for both healthcare professionals and service users.

### 4.5. Demographic Differences

In this review, participants were 32% Caucasian and 66% female. This is supported by existing research that reports the demand for healthcare services is greater in women living with overweight and obesity [103]. Women tend to report higher EES than men [104]. However, a further study found that men with EE were almost three times more likely be overweight than women [105]. Black adults are at higher risk of developing obesity than Caucasians, and Chinese adults are at lower risk of developing obesity [106]. Whilst there were a mix of genders and ethnicities within this review, future research should focus on a wider demographic population to increase the generalizability of the findings of this review.

### 4.6. Strengths and Limitations

#### 4.6.1. Strengths

The strengths of this review included broad and clear inclusion criteria that were designed through public and patient involvement and engagement. The authors did not limit the searches to RCTs in the way that Chew et al. (2022) [25] designed their review. This allowed for the inclusion of non-randomised intervention studies and single group designs. Furthermore, the changes in weight and EE scores were converted to percentages due to the range in baseline means, and the wide variety of tools used to measure EE. This allowed the authors to include additional studies and improved the interpretation of results. Furthermore, there were a mix of participant ages, genders, and ethnicities in the included participants.

#### 4.6.2. Limitations

There were several limitations to this systematic review and meta-analysis. First, there was heterogeneity in measurement tools and interventions. The studies used multiple tools to measure EE (*n* = 11). Determining the validity of EE questionnaires is vital, given that these measures are more feasible for settings that do not allow observation of food intake and there is known measurement invariance in EE measures (namely DBEQ) by gender, age and BMI; therefore, it may be useful to segregate these groups in future analyses [107]. There was also wide variation in included interventions, and many were multi-component interventions, making it was difficult to fully understand the active components. Second, the statistical heterogeneity of the studies included in the meta-analysis and the significant publication bias in both meta-analyses reduce the validity of the overall effect size for weight and EE. The effect sizes were therefore adjusted using the Trim and Fill procedure to estimate an unbiased effect size. Third, the primary focus of several studies was on weight or BMI which can take longer to show significant changes over a relatively short intervention. Finally, whilst we reported on the risk of bias of individual studies and considered the impact of heterogeneity and publication bias in the meta-analysis, a formal GRADE [108] evaluation was not undertaken for this review. Future research should therefore consider a GRADE evaluation prior to making practice recommendations.

## 5. Conclusions

This comprehensive systematic review and meta-analysis found that psychological interventions to address EE showed some promise in reducing EE and promoting a small amount of weight loss in adults living with overweight or obesity. CBT showed the most promise for reducing weight and improving EE in the sub analyses. The review identified a wide range of available psychological interventions to address EE in this population, and wide variation in the available tools to assess for EE. A consensus is therefore required amongst healthcare professionals and academics regarding the most feasible and acceptable tool to identify EE in weight management practice settings. An important first step in weight management practice would be the development of an agreed pathway for EE in adults with overweight or obesity. Further research is required involving participants from a wide range of ethnic backgrounds, genders, and/or socio-economic statuses. Future research should focus on the long-term effectiveness and acceptability of interventions that address EE in adults with overweight or obesity and to better understand the active components and mechanisms of change in effective interventions.

## Figures and Tables

**Table 1:** Search Terms.

Population	Intervention	Outcome
obes * OR overweight OR weight OR BMI OR body mass index OR waist circumference AND Adult * OR over 18	Mindful * OR mindful eat * OR Emotional Eating OR cognitive behavio * OR Behavio * change OR binge eat * OR comfort eat * OR self-help OR food addiction OR Acceptance and Commitment Therapy OR ACT AND Intervention * OR treatment *	Weight loss OR weight reduction OR lose weight OR eating control

* Truncation used to search.

## Supplementary Materials

The following supporting information can be downloaded at: https://www.mdpi.com/article/10.3390/ijerph20032722/s1, Table S1: Systematic Review PRISMA checklist; Table S2: Results of Included Studies; Table S3: Hierarchy of Inclusion and Exclusion Criteria; Table S4: Critical Appraisal Tool For Randomised Control Trials [61]; Table S5: JBI Checklist for Quasi-Experimental Studies (Non-Randomised Experimental Studies) [62].
