# Supplementary material for: Emotional Eating Interventions for Adults Living with Overweight or Obesity: A Systematic Review and Meta-Analysis
Source: Int J Environ Res Public Health. 2023 Feb 3;20(3):2722. doi: 10.3390/ijerph20032722 (PMC9915727; doi:10.3390/ijerph20032722)
Supplement: Supplementary file 1 [file ijerph-20-02722-s001.zip › ijerph-2187745-supplementary.pdf]

Emotional Eating interventions for adults living with overweight or obesity: A systematic review and meta-analysis

Jo Smith, Xiao Qi Ang, Emma L. Giles, and Gemma Traviss-Turner

Supplementary Materials

Supplementary Table S1: Systematic Review PRISMA checklist.

Section and Topic	Item #	Checklist item	Location where item is reported (section)
**TITLE**			
Title	1	Identify the report as a systematic review.	Title
**ABSTRACT**			
Abstract	2	See the PRISMA 2020 for Abstracts checklist.	Abstract
**INTRODUCTION**			
Rationale	3	Describe the rationale for the review in the context of existing knowledge.	1. Introduction
Objectives	4	Provide an explicit statement of the objective(s) or question(s) the review addresses.	1. Introduction: Aim and research questions
**METHODS**			
Eligibility criteria	5	Specify the inclusion and exclusion criteria for the review and how studies were grouped for the syntheses.	2.1 Eligibility criteria, search strategy and selection of studies

Section and Topic	Item #	Checklist item	Location where item is reported (section)
Information sources	6	Specify all databases, registers, websites, organisations, reference lists and other sources searched or consulted to identify studies. Specify the date when each source was last searched or consulted.	2.1 Eligibility criteria, search strategy and selection of studies
Search strategy	7	Present the full search strategies for all databases, registers and websites, including any filters and limits used.	2.1 Eligibility criteria, search strategy and selection of studies
Selection process	8	Specify the methods used to decide whether a study met the inclusion criteria of the review, including how many reviewers screened each record and each report retrieved, whether they worked independently, and if applicable, details of automation tools used in the process.	FIGURE 1: PRISMA flow chart
Data collection process	9	Specify the methods used to collect data from reports, including how many reviewers collected data from each report, whether they worked independently, any processes for obtaining or confirming data from study investigators, and if applicable, details of automation tools used in the process.	FIGURE 1: PRISMA flow chart
Data items	10a	List and define all outcomes for which data were sought. Specify whether all results that were compatible with each outcome domain in each study were sought (e.g. for all measures, time points, analyses), and if not, the methods used to decide which results to collect.	2.1 Eligibility criteria, search strategy and selection of studies
	10b	List and define all other variables for which data were sought (e.g. participant and intervention characteristics, funding sources). Describe any assumptions made about any missing or unclear information.	2.1 Eligibility criteria, search strategy and selection of studies

Section and Topic	Item #	Checklist item	Location where item is reported (section)
Study risk of bias assessment	11	Specify the methods used to assess risk of bias in the included studies, including details of the tool(s) used, how many reviewers assessed each study and whether they worked independently, and if applicable, details of automation tools used in the process.	2.3 Critical appraisal
Effect measures	12	Specify for each outcome the effect measure(s) (e.g. risk ratio, mean difference) used in the synthesis or presentation of results.	Supplementary Table S2: Results of Included Studies
Synthesis methods	13a	Describe the processes used to decide which studies were eligible for each synthesis (e.g. tabulating the study intervention characteristics and comparing against the planned groups for each synthesis (item #5)).	2.1 Eligibility criteria, search strategy and selection of studies
	13b	Describe any methods required to prepare the data for presentation or synthesis, such as handling of missing summary statistics, or data conversions.	2.4 Synthesis of results and analytical strategy
	13c	Describe any methods used to tabulate or visually display results of individual studies and syntheses.	Figure 2: Forest Plot for percentage weight change<br>Figure 3: Funnel Plot for effect size against standard error for percentage

Section and Topic	Item #	Checklist item	Location where item is reported (section)
			weight change<br>Figure 4: Forest Plot for effect size of percentage change in EE<br>Figure 5: Funnel Plot for effect size against standard error for percentage change in EE score
	13d	Describe any methods used to synthesize results and provide a rationale for the choice(s). If meta-analysis was performed, describe the model(s), method(s) to identify the presence and extent of statistical heterogeneity, and software package(s) used.	3.4 Meta-Analysis<br>3.4.1 Weight<br>3.4.2 EE Score
	13e	Describe any methods used to explore possible causes of heterogeneity among study results (e.g. subgroup analysis, meta-regression).	3.4 Meta-Analysis<br>3.4.1 Weight<br>3.4.2 EE Score
	13f	Describe any sensitivity analyses conducted to assess robustness of the synthesized results.	3.4 Meta-Analysis<br>3.4.1 Weight<br>3.4.2 EE Score

Section and Topic	Item #	Checklist item	Location where item is reported (section)
Reporting bias assessment	14	Describe any methods used to assess risk of bias due to missing results in a synthesis (arising from reporting biases).	3.5 Methodological quality<br>CASP and JBI
Certainty assessment	15	Describe any methods used to assess certainty (or confidence) in the body of evidence for an outcome.	3.4 Meta-Analysis<br>3.4.1 Weight<br>3.4.2 EE Score
**RESULTS**			
Study selection	16a	Describe the results of the search and selection process, from the number of records identified in the search to the number of studies included in the review, ideally using a flow diagram.	FIGURE 1: PRISMA flow chart<br>Supplementary Table S3
	16b	Cite studies that might appear to meet the inclusion criteria, but which were excluded, and explain why they were excluded.	FIGURE 1: PRISMA flow chart
Study characteristics	17	Cite each included study and present its characteristics.	Table 2: Summary of Included Studies
Risk of bias in studies	18	Present assessments of risk of bias for each included study.	3.5 Supplementary Tables S2, S4 and S5.
Results of	19	For all outcomes, present, for each study: (a) summary statistics for each group (where appropriate) and (b) an effect estimate	Supplementary

Section and Topic	Item #	Checklist item	Location where item is reported (section)
individual studies		and its precision (e.g. confidence/credible interval), ideally using structured tables or plots.	Table S2: Results of Included Studies
Results of syntheses	20a	For each synthesis, briefly summarise the characteristics and risk of bias among contributing studies.	Supplementary Table S2: Results of Included Studies
	20b	Present results of all statistical syntheses conducted. If meta-analysis was done, present for each the summary estimate and its precision (e.g. confidence/credible interval) and measures of statistical heterogeneity. If comparing groups, describe the direction of the effect.	3.4 Meta-Analysis<br>3.4.1 Weight<br>3.4.2 EE Score
	20c	Present results of all investigations of possible causes of heterogeneity among study results.	3.4 Meta-Analysis<br>3.4.1 Weight<br>3.4.2 EE Score
	20d	Present results of all sensitivity analyses conducted to assess the robustness of the synthesized results.	3.4 Meta-Analysis<br>3.4.1 Weight<br>3.4.2 EE Score
Reporting biases	21	Present assessments of risk of bias due to missing results (arising from reporting biases) for each synthesis assessed.	3.4.2 EE Score
Certainty of evidence	22	Present assessments of certainty (or confidence) in the body of evidence for each outcome assessed.	3.4 Meta-Analysis

Section and Topic	Item #	Checklist item	Location where item is reported (section)
			3.4.1 Weight<br>3.4.2 EE Score
**DISCUSSION**			
Discussion	23a	Provide a general interpretation of the results in the context of other evidence.	4. Discussion<br>4.1 Intervention effects on weight (Kg)<br>4.2 Interventions for improving EE<br>4.3 Impact of style and setting of intervention delivery<br>4.4 Screening tools for EE<br>4.5 Demographic differences
	23b	Discuss any limitations of the evidence included in the review.	4.5.2 Limitations
	23c	Discuss any limitations of the review processes used.	4.5.2 Limitations

Section and Topic	Item #	Checklist item	Location where item is reported (section)
	23d	Discuss implications of the results for practice, policy, and future research.	8. Conclusion and future directions
**OTHER INFORMATION**			
Registration and protocol	24a	Provide registration information for the review, including register name and registration number, or state that the review was not registered.	2. Materials and Methods
	24b	Indicate where the review protocol can be accessed, or state that a protocol was not prepared.	2. Materials and Methods
	24c	Describe and explain any amendments to information provided at registration or in the protocol.	n/a
Support	25	Describe sources of financial or non-financial support for the review, and the role of the funders or sponsors in the review.	Funding Declaration
Competing interests	26	Declare any competing interests of review authors.	Conflicts of Interest Statement
Availability of data, code and other materials	27	Report which of the following are publicly available and where they can be found: template data collection forms; data extracted from included studies; data used for all analyses; analytic code; any other materials used in the review.	Reference list

Supplementary Table S2: Results of Included Studies

Authors, Date, Intervention Name and Country	Study Design and Population	Intervention(s)	Weight and EE Outcomes (percentage change)	Quality Assessment Tool, Score and Percentage
Afari et al. (2019) [30]<br>MOVE + ACT<br>USA	**Design:** RCT<br>**Sample size:** n = 88 (85 completers)<br>**Mean age:** 57.3 years (SD:9.9).<br>**Gender:** 76.1% male,<br>**Ethnicity:** 70.5% Caucasian, 17% African American, and 12% Hispanic<br>**Mean weight**: 112.7Kg (SD: 26.3)<br>**Mean BMI**: 37.2 kg/m^2 (SD:7.0)	**Interventions:**<br>• Acceptance and Commitment Therapy (ACT).<br>• Behavioural Weight Loss (BWL)<br>**Both length of interventions:** 8 weeks in person, 90 days by telephone<br>**Settings:** In person and by telephone<br>**Delivered by:** (ACT) **-** A full-time staff psychologist; 2 psychology postdoctoral fellows; a psychology master's student; and ACT therapists. (BWL) - A full-time staff psychologist; a psychology postdoctoral fellow and a psychology master's student.	**Percentage weight change:**<br>ACT (n=43): -0.44% (95%CI: -2.92 to 2.04)<br>BWL (n=42): -0.62% (95%CI: -3.25 to -2.02)<br><br>**Percentage change in Emotional Eating:**<br>Measured by the Dutch Eating Behaviour Questionnaire [66]<br>ACT (n=43): -6.67% (95%CI: -43.95 to 30.62)<br>BWL (n=42): -13.3% (95%CI: -53.14 to 26.48)	**Tool:** CASP RCT<br>**Score:** 11<br>**Percentage:** 100
Annesi et al. (2016) [31]<br>USA	**Design:** Individually Randomized Group Treatment Trial<br>**Sample size:** n = 103<br>**Mean age:** 47.8 years (SD: 7.9)<br>**Gender:** 100% female<br>**Ethnicity:** 84 % White, 12 % African American, and 4 % other<br>**Mean weight**: 95.58Kg (SD:11.08)<br>**Mean BMI**: 35.4Kg/m^2 (SD: 3.3)	**Interventions:**<br>• Mindfulness (Personal contact group or self-help).<br>**Length of the interventions:** 6 months<br>**Settings: G**roup (personal contact group or self-help) and in-person (personal contact)<br>**Delivered by:** A trained and nationally certified wellness professional.	**Percentage weight change:**<br>Self-help (n=50): -1.94% (95%CI: -1.91 to 5.79)<br>Personal contact (n=53): -5.95% (95%CI: -11.55 to -0.34)<br><br>**Percentage change in Emotional Eating:**<br>Measured by the Emotional Eating Scale (EES) [67]<br>Self-help (n=50): -35.47% (95%CI: -54.05 to -16.88)	**Tool:** CASP RCT<br>**Score:** 9<br>**Percentage:** 81.8

			Personal contact (n=53): -21.40% (95%CI: -38.92 to -3.88)	
Annesi (2019) [32]<br>USA	**Design:** Individually Randomized Group Treatment Trial<br>**Sample size:** n = 152<br>**Mean age:** 48.6 years (SD:7.0)<br>**Gender:** 100% female<br>**Ethnicity:** 80% White, 15% Black, 5% other<br>**Mean weight**: Not reported<br>**Mean BMI**: 35.3 Kg/m² (SD:3.2)	**Interventions:**<br>• Group 1: Behavioural Weight Loss (BWL)<br>• Group 2: Behavioural Weight Loss (BWL)<br>• + Cognitive Behavioural Treatment (CBT)<br>• Group 3: Behavioural Weight Loss (BWL)<br>• + Cognitive Behavioural Treatment (CBT).<br>**Length of interventions:**<br>Group 1: 28 weeks<br>Group 2: 58 weeks<br>Group 3: 99 weeks<br>**Setting:**<br>Group 1: phone call<br>Group 2: 1:1 support in person<br>Group 3: phone call and 1:1- support, in person<br>**Delivered by:** All intervention were delivered by the authors**.**	**Percentage weight change:**<br>Only BMI reported.<br><br>**Percentage change in Emotional Eating:**<br>Measured by the Emotional Eating Scale (EES) [67]<br>Group 1 (n=52): -23.69% (95%CI: -41.56 to -5.81)<br>Group 2 (n=52): -31.45% (95%CI: -49.69 to -13.20)<br>Group 3 (n=48): -23.96% (95%CI: -42.46 to -5.45)	**Tool:** CASP RCT<br>**Score:** 10<br>**Percentage:** 90.9
Bacon et al. (2005) [33]<br>Health at Every Size<br>USA	**Design:** Individually Randomized Group Treatment Trial<br>**Sample size:** n = 35 completers at post-intervention.<br>**Mean age:** HAES: 40.4 (SD:4.4); Diet: 41.4 years (SD:3.0)<br>**Gender:** 100% female<br>**Ethnicity:** Not reported	**Interventions:**<br>• Diet Group (BWL)<br>• Health at Every Size (HAES) Group (Acceptance-based)<br>**Length of interventions:** 6 months<br>**Setting:** Group<br>**Delivered by:** The Diet Group program was taught by an experienced Registered Dietitian. The Health at Every Size	**Percentage weight change:**<br>Diet Group (n=16): -4.35% (95%CI: -9.48 to +0.78)<br>HAES (n=19): +0.69% (95%CI: -2.36 to +3.75)<br><br>**Percentage change in Emotional Eating:**	**Tool:** CASP RCT<br>**Score:** 10<br>**Percentage:** 90.9

	**Mean weight**: Diet: 101.2Kg (SD:13.8); HAES: 101.1Kg (SD:13.3)<br>**Mean BMI**: Diet: 36.7 Kg/m² (SD:4.2); HAES: 35.9Kg/m² (SD:4.6)	Group was facilitated by a counsellor who had conducted educational and psychotherapeutic workshops.	Measured by the Eating Inventory [69]<br>Diet Group (n=16): - 31.15% (95%CI: -59.59 to -2.70)<br>HAES (n=19): -37.19% (95%CI: -66.13 to -8.25)	
Carbine et al. (2021) [34]<br>USA	**Design:** RCT<br>**Sample size:** n = 100<br>**Mean age:** 28.05 years (SD:7.56)<br>**Gender:** 53% Female<br>**Ethnicity:** 81% Caucasian, 14% Hispanic, 5% other<br>**Mean weight**: 95.95Kg (SD:18.65)<br>**Mean BMI**: 32.48Kg/m² (SD:5.36)	**Interventions:**<br>• Food Specific ICT (i.e., inhibiting responses to high-calorie foods)<br>• Generic ICT (i.e., inhibiting responses to everyday items)<br>**Length of interventions:** 4 weeks<br>**Settings:** In person<br>**Both delivered by:** The authors**.**	**Percentage weight change:**<br>Food Specific ICT (n=52): -0.05% (95%CI: -2.51 to 2.40)<br>Generic ICT (n=48): +0.16% (95%CI: -2.51 to 2.75)<br><br>**Percentage change in Emotional Eating:**<br>Measured by the Dutch Eating Behaviour Questionnaire [66]<br>Food Specific ICT (n=52): -0.72% (95%CI: -35.84 to 34.40)	**Tool:** CASP RCT<br>**Score:** 11<br>**Percentage:** 100
Carpenter et al. (2019) [35]<br>Mind Your Weight<br>USA	**Design:** Individually Randomized Group Treatment Trial, pilot study<br>**Sample size:** n = 75<br>**Mean age:** 47.3 years (SD:10.0)<br>**Gender:** 92% Female<br>**Ethnicity:** 65.3% White, 26.7% black, 6.7% Hispanic, 1.3% Asian<br>**Mean weight**: Not reported<br>**Mean BMI**: 31.5Kg/m² (SD:2.3)	**Interventions:**<br>• Mindfulness weight loss program (Mind Your Weight)<br>• Behavioural weight loss program (Weight Talk™)<br>**Length of interventions:** 6-months<br>**Settings:** Telephone based counselling<br>**Both delivered by:** Health Coaches and Registered Dietitians	**Percentage weight change:**<br>Insufficient data reported to calculate.<br><br>**Percentage change in Emotional Eating:**<br>Measured by the Mindful Eating Questionnaire [70]<br>Mind Your Weight (n=50): -22.73% (95%CI: -67.92 to +22.47)<br>Weight TalkTM (n=25): -4.35% (95%CI: -44.60 to +35.91)	**Tool:** CASP RCT<br>**Score:**11<br>**Percentage:** 100
Chung et al. (2016) [36]<br>USA	**Design:** Single-grouped design longitudinal study<br>**Sample size:** n = 22	**Intervention:**<br>• Mindful Eating<br>**Length of intervention:** 12 weeks	**Percentage weight change:**<br>Mindful eating (n=22): -0.98% (95%CI: -4.45 to 2.48)	**Tool:** JBI Quasi-experimetal<br>**Score:** 5

	**Mean age:** 50.14 years (SD:9.0)<br>**Gender:** 100% Female<br>**Ethnicity:** 100% African American<br>**Mean weight**: 92.44Kg (SD: 16.05)<br>**Mean BMI**: 35.13Kg/m^2 (SD: 3.97)	**Setting:** Group, in person<br>**Delivered by:** Registered Dietitian	**Percentage change in Emotional Eating:**<br>Measured by the Mindful Eating Questionnaire [70]<br>Mindful eating (n=22): -1.05% (95%CI: -37.19 to 35.10)	**Percentage:** 83.3
Daubenmier et al. (2016) [37]<br>SHINE<br>USA	**Design:** RCT<br>**Sample size:** n = 194<br>**Mean age:** 47.5 years (SD:12.7)<br>**Gender:** 80% Female<br>**Ethnicity:** 59.3% European, 12.9% African, 9.8% Asian/Pacific Islander, 11.9% Latina/Latino, 1% Native American, 5.1% other<br>**Mean weight:** 97.2Kg (SD: 14.4)<br>**Mean BMI:** 35.5Kg/m^2 (SD: 3.6)	**Interventions:**<br>• Mindfulness-based weight loss intervention<br>• Behavioural Weight Loss as active control group<br>**Length of interventions:** Both interventions included 16 sessions lasting 2 to 2.5 h (12 weekly, 3 bi-weekly, and 1 monthly) and one all-day session (6.5 and 5 h in the mindfulness and control interventions, respectively) over 5.5 months.<br>**Setting:** Group and in person<br>**Delivered by:** Registered Dietitians, with the Mindfulness being delivered by Mindfulness Meditation Instructors.	**Percentage weight change:**<br>Mindfulness (n=100): -4.61% (95%CI: -9.58 to 0.36)<br>Behavioural Weight Loss (n=94): -3.41% (95%CI: -7.91 to 1.11)<br><br>**Percentage change in Emotional Eating:**<br>Not measured.	**Tool:** CASP RCT<br>**Score:**11<br>**Percentage:** 100
Forman et al. (2013) [38]<br>Mind your Health<br>USA	**Design:** RCT<br>**Sample size:** n = 128 (99 completers post-intervention)<br>**Mean age:** 45.69 years (SD:12.81)<br>**Gender:** Not reported<br>**Ethnicity:** 62.3% Caucasian, 24.6% African American, 1.6% Asian, 3.8% Hispanic<br>**Mean weight**: Not reported	**Interventions:**<br>• Acceptance-based Behavioural Treatment - ABT<br>• Standard Behavioural Treatment (SBT)<br>**Length of intervention:** 40 weeks<br>**Settings:** Group, in person<br>**Delivered by:** Novice trainers (i.e., advanced doctoral students who had	**Percentage weight change:**<br>Insufficient data reported to calculate.<br><br>**Percentage change in Emotional Eating:**<br>Measured by the Emotional Eating Scale (EES) [67]	**Tool:** CASP RCT<br>**Score:** 9<br>**Percentage:** 81.8

	**Mean BMI**: 34.10Kg/m² (SD:3.64)	received specific training in both ABT and SBT) or expert trainers (i.e., clinical psychologists with experience administering behavioural weight control interventions).	ABT (n=59): -22.94% (95%CI: -35.01 to -10.87)<br>SBT (n=40): -14.22% (95%CI: -24.52 to -3.91)	
Frayn et al. (2020) [23]<br>Switzerland	**Design:** Single-group design<br>**Sample size:** n = 32<br>**Mean age:** 46.71 years (SD:13.43)<br>**Gender:** 87.5% Female, 12.5% Male<br>**Ethnicity:** 78.1% Caucasian, 3.1% Middle eastern, 3.1% Black, 3.1% Hispanic, 12.5% Others<br>**Mean weight**: Not reported<br>**Mean BMI**: 33.13Kg/m² (SD:5.40)	**Intervention:**<br>• Acceptance and Commitment Therapy (ACT)<br>**Length of intervention:** 1 day<br>**Setting:** Group, in person<br>**Delivered by**: A PhD candidate in clinical psychology who had training in ACT.	**Percentage weight change:**<br>Only BMI reported.<br><br>**Percentage change in Emotional Eating:**<br>Measured by the Dutch Eating Behaviour Questionnaire (DEBQ) [66]<br>ACT (n=32): -20.72% (95%CI: -59.91 to 18.48)	**Tool:** JBI Quasi-experimetal<br>**Score:** 5<br>**Percentage:** 83.3
Goldbacher et al. (2016) [24]<br>USA	**Design:** Individually Randomized Group Treatment Trial<br>**Sample size:** n = 79<br>**Mean age:** 45.6 years (SD:10.5)<br>**Gender:** 95% Female, 5% Male<br>**Ethnicity:** 80% African American, 11% White, 4% Hispanic, 5% Other<br>**Mean weight**: 97.9Kg (SD:12.2)<br>**Mean BMI**: 36.2Kg/m² (SD:4.1)	**Interventions:**<br>• Behavioural Weight Loss Treatment (SBT)<br>• Enhanced Behavioural Treatment (EBT)<br>**Length of intervention:** 20 weeks<br>**Setting:** Group, in person<br>**Both delivered by:** Masters- and doctoral-level clinicians.	**Percentage weight change:**<br>SBT (n=39): -6.75% (95%CI: -12.11 to -1.39)<br>EBT (n=40): -6.38% (95%CI: -11.51 to -1.24)<br><br>**Percentage change in Emotional Eating:**<br>Measured by the Emotional Eating Scale (EES) [67]<br>SBT (n=39): -42.89% (95%CI: -58.08 to -27.71)<br>EBT (n=40): -39.63% (95%CI: -55.16 to -24.10)	**Tool:** CASP RCT<br>**Score:** 11<br>**Percentage:** 100
Hanson et al. (2019) [39]<br>UK	**Design:** Single-group design<br>**Sample size:** n = 53 (33 completers)	**Interventions:**<br>• Mindfulness<br>• Compared to control group	**Percentage weight change:**<br>Mindfulness (n=33): -2.38% (95%CI: -5.86 to 1.11)	**Tool:** JBI Quasi-experimetal<br>**Score:** 5

	**Mean age:** 44.4 years (SD:11.0)<br>**Gender:** 78.8% Female<br>**Ethnicity:** Not reported<br>**Mean weight**: 126.3Kg (SD:36.1)<br>**Mean BMI**: 46.5kg/m^2 (SD:8.0)	**Length of intervention:** 8 weeks<br>**Setting:** Group, in person<br>**Delivered by:** A team of specialist dietitians, psychologists, and physicians.	**Percentage change in Emotional Eating:**<br>Measured by the Whole Person Integrated Eating Questionnaire (WPIEQ) Emotional Eating Subscale [75]<br>Mindfulness (n=33): -47.83% (95%CI: -66.88 to -28.78)	**Percentage:** 83.3
Hawkins et al. (2021) [40]<br>POWER-UP<br>USA	**Design:** Single group design<br>**Sample size:** n = 48<br>**Mean age:** 43.58 years (SD:1.50)<br>**Gender:** 85.4% Female<br>**Ethnicity:** Not reported<br>**Mean weight**: 101.96Kg (SD:2.09)<br>**Mean BMI**: 36.83kg/m^2 (SD:0.63)	**Intervention:**<br>• Acceptance-based Behavioural Therapy - ABT<br>**Length of intervention:** 23 weeks<br>**Setting:** Group, in person<br>**Delivered by:** A nurse trained in BWL and a clinical psychologist.	**Percentage weight change:**<br>ABT (n=48): -5.04% (95%CI: -10.12 to 0.03)<br><br>**Percentage change in Emotional Eating:**<br>Measured by the Emotional Eating Scale (EES) [67]<br>ABT (n=48): -32.67% (95%CI: -51.64 to -13.69)	**Tool:** JBI Quasi-experimetal<br>**Score:** 5<br>**Percentage:** 83.3
Hepdurgun et al. (2020) [41]<br>Turkey	**Design:** Individually Randomized Group Treatment Trial<br>**Sample size:** n = 51 in the intervention group.<br>**Mean age:** 40.1 years (SD:9.96)<br>**Gender:** 80.4% Female, 19.6% Male<br>**Ethnicity:** Not reported<br>**Mean weight**: 87.66Kg (SD:14.33)<br>**Mean BMI**: 32.17Kg/m^2 (SD:4.26)	**Interventions:**<br>• Internet-Based Behavioural Therapy - IBT<br>• Compared to routine care (healthy eating and physical activity information) by email.<br>**Length of intervention:** 8-weeks<br>**Setting:** Internet based (with training beforehand in person)<br>**Delivered by:** Online	**Percentage weight change:**<br>IBT (n=51): -3.32% (95%CI: -8.14 to +1.50)<br><br>**Percentage change in Emotional Eating:**<br>Not measured.	**Tool:** CASP RCT<br>**Score:** 10<br>**Percentage:** 90.9

Hunot-Alexander et al. (2021) [42]<br>UK	**Design:** Single-group design with qualitative evaluation<br>**Sample size:** n = 37 (weight data reported for 32)<br>**Mean age:** 48.3 years (SD:10.9)<br>**Gender:** 93.8% Female, 6.3% Male<br>**Ethnicity:** 90.6% White, 9.4% Non-white<br>**Mean weight**: Not reported<br>**Mean BMI**: 34.3Kg/m^2 (SD:6.9)	**Intervention:**<br>• Appetitive Trait Tailored Intervention (ATTI)<br>**Length of intervention:** 8 weeks<br>**Setting:** Not specified<br>**Delivered by:** A PhD student.	**Percentage weight change:**<br>ATTI (n=32): -1.42% (95%CI: -5.13 to 2.29)<br><br>**Percentage change in Emotional Eating:**<br>Not measured.	**Tool:** JBI Quasi-experimetal<br>**Score:** 2<br>**Percentage:** 33.3
Kearney et al. (2012) [43]<br>USA	**Design:** Single Group Design<br>**Sample size:** n = 48 (38 completers post-intervention)<br>**Mean age:** 49 years (SD:10.7)<br>**Gender:** 87.5% Male<br>**Ethnicity:** 85.4% White, 4.2% Black, 6.3% Hispanic, 4.2% Asian/Pacific-Islander/Native American<br>**Mean weight**: 95.1Kg (SD not reported)<br>**Mean BMI**: 29.4Kg/m^2 (SD not reported)	**Intervention**:<br>• Mindfulness-based stress reduction (MBSR).<br>**Length of intervention**: 2 months<br>**Setting:** Group, in person<br>**Delivered by:** Instructors who met professional guidelines for teaching MBSR	**Percentage weight change:**<br>MBSR (n=38): +0.84% (95%CI: -2.34 to 4.02)<br><br>**Percentage change in Emotional Eating:**<br>Measured by the Three Factor Eating Questionnaire (TFEQ) [68]<br><br>MBSR (n=38): -2.94% (95%CI: -27.28 to -14.40)	**Tool:** JBI Quasi-experimetal<br>**Score:** 5<br>**Percentage:** 83.3
Keränen et al. (2009) [44]<br>LITE<br>Finland	**Design:** Individually Randomized Group Treatment Trial<br>**Sample size:** n = 20 completers in the intervention group<br>**Mean age:** 52 years (SD: 7.0)<br>**Gender:** 25% Male<br>**Ethnicity:** Not reported<br>**Mean weight**: 98.0Kg (SD: 18.0)	**Interventions**:<br>• Intensive Counselling (with components of EE)<br>• Compared to Short-term Counselling (no components ofEE)<br>**Length of interventions**: 20-weeks<br>**Setting:** Group and personal sessions<br>**Delivered by:** A Clinical Nutritionist	**Percentage weight change:**<br>Intensive counselling (n=20: -5.10% (95%CI: -10.53 to +0.33)<br><br>**Percentage change in Emotional Eating:**<br>Measured by the Three Factor Eating Questionnaire (TFEQ) [68]	**Tool:** CASP RCT<br>**Score:** 11<br>**Percentage:** 100

	**Mean BMI**: 35.0Kg/m² (SD: 5.0)		Insufficient data reported to calculate.	
Kidd et al. (2013) [45]<br>USA	**Design:** Single Group Design<br>**Sample size:** n = 12<br>**Mean age:** 51.8 years (SD: 9.1)<br>**Gender:** 100% Female<br>**Ethnicity:** 58.3% African American, 41.7% White<br>**Mean weight**: 119.8Kg (SD: 16.9)<br>**Mean BMI**: 44.7Kg/m² (SD: 6.9)	**Intervention**:<br>• Mindful Eating<br>**Length of intervention**: 8-weeks<br>**Setting:** Face-to-face<br>**Delivered by:** The Research Team	**Percentage change in weight:**<br>Mindful Eating (n=12): -0.58% (95%CI: -3.30 to +2.13)<br><br>**Percentage change in Emotional Eating:**<br>Measured by the Mindful Eating Questionnaire (MEQ) [70]<br>Mindful Eating (n=12): -3.88% (95%CI: -10.60 to +2.84)	**Tool:** JBI Quasi-experimetal<br>**Score:** 4<br>**Percentage:** 66.7
Kim et al. (2021) [46]<br>Healthy Life Plan<br>Korea	**Design:** RCT<br>**Sample size:** n = 583 (369 completers post-intervention)<br>**Mean age:** 53.68 years (SD:10.12) for the IG and 53.94 years (SD: 10.18) for the MG.<br>**Gender:** 61.6% Female, 38.4% Male<br>**Ethnicity:** Not reported<br>**Mean weight**: Not reported<br>**Mean BMI**: 28.03Kg/m² (SD:2.83) for the IG and 28.20Kg/m² (SD:3.15) for the MG.	**Intervention:**<br>• Intensive intervention group (IG). Received a multi-component intervention to reduce abdominal obesity, by mainly focusing on dietary attitude and dietary behaviour change, and a minimal information intervention.<br>• Minimal information intervention group [83]. Received a brief explanation of health status and a simple recommendation for a lifestyle change<br>**Length of intervention:** 6 months<br>**Setting:** Group, in person<br>**Delivered by:** Trained Clinical Nutritionists	**Percentage weight change:**<br>Only BMI reported.<br><br>**Percentage change in Emotional Eating:**<br>Measured by the Dutch Eating Behaviour Questionnaire (DEBQ) [66]<br>Intensive intervention group (IG) (n=173): -3.24% (95%CI: -41.81 to 35.32)<br>Minimal information intervention group (n=196): -4.93% (95%CI: -43.88 to 34.02)	**Tool:** CASP RCT<br>**Score:** 11<br>**Percentage:** 100
Lillis et al. (2016) [47]	**Design:** RCT<br>**Sample size:** n = 162	**Interventions:**	**Percentage weight change:**	**Tool:** CASP RCT<br>**Score:** 11

USA	**Mean age:** 50.2 years (SD: 10.9)<br>**Gender:** 85% Female, 15% Male<br>**Ethnicity:** 5% Black/African American, 6% Hispanic, 1% Asian, 88% Caucasian<br>**Mean weight**: 102.3Kg (SD:17.4)<br>**Mean BMI**: 37.6Kg/m² (SD:5.3)	• Standard Behavioural Treatment (SBT)<br>• Acceptance-Based Behavioural Intervention (ABBI)<br>**Length of interventions:** 24 months<br>**Setting:** Group, in person<br>**Delivered by:** A Ph.D. psychologist; a Ph.D. exercise physiologist; and a master's level nutritionist.	SBT (n=81): -2.59% (95%CI: -6.59 to 1.41)<br>ABBI (n=81): -4.19% (95%CI: -8.87 to 0.50)<br><br>**Percentage change in Emotional Eating:**<br>Measured by the internal disinhibition subscale of the Eating Inventory [69]<br>SBT (n=81): -23.61% (95%CI: -57.10% to 9.88%)<br>ABBI (n=81): -28.71% (95%CI: -63.05 to 5.63)	**Percentage:** 100
Malkina-Pykh (2012) [48]<br>Russia	**Design:** RCT<br>**Sample size:** n = 104 (58 completers at post-intervention)<br>**Mean age:** 37.6 years (SD:6.7)<br>**Gender:** 69% Female. 31% Male<br>**Ethnicity:** Not reported<br>**Mean weight**: Not reported<br>**Mean BMI**: 34.4Kg/m² (SD:6.6) for CBT group and 34.6Kg/m² (SD: 6.1) for the CBT and RMT group.	**Intervention:**<br>• Cognitive Behavioural Therapy (CBT) +/-<br>• Rhythmic Movement Therapy (RMT). This was added for half of participants who showed no improvement with CBT after 6 months (n=30). The remaining 28 participants who did not respond to CBT after 6 months continued with CBT.<br>**Length of intervention**: 24 bi-weekly sessions (48 weeks)<br>**Setting:** Individual, in person<br>**Delivered by:** Psychologists trained in CBT	**Percentage weight change:**<br>Only BMI reported.<br><br>**Percentage change in Emotional Eating:**<br>Measured by the Dutch Eating Behaviour Questionnaire (DEBQ) [66]<br>CBT only (n=28): -0.88% (95%CI: -34.07 to 32.31)<br>CBT and RMT (n=30): -19.24% (95%CI: -58.87 to 20.39)	**Tool:** CASP RCT<br>**Score:** 10<br>**Percentage:** 90.9
Manzoni et al. (2009) [49]<br>Italy	**Design:** RCT<br>**Sample size:** n = 40 in the two intervention groups.	**Intervention:**<br>• Relaxation training – traditional (imagination condition)	**Percentage weight change:**<br>Imagination condition (n=20: -6.10% (95%CI: -11.67 to -0.53)	**Tool:** CASP RCT<br>**Score:** 9<br>**Percentage:** 81.8

	**Mean age:** Not reported<br>**Gender:** 100% Female<br>**Ethnicity:** Not reported<br>**Mean weight**: Imagination condition: 104.9Kg (SD: not reported); Virtual reality condition: 110.6Kg (SD: not reported)<br>**Mean BMI**: Not reported	• Relaxation training (virtual reality condition)<br>• Compared to standard hospital-based care<br>**Length of intervention**: 5 weeks<br>**Setting:** Inpatient ward.<br>**Delivered by:**<br>Two licensed clinical psychologists and one licensed psychotherapist under the supervision of a senior psychotherapist.	Virtual reality condition (n=20): -0.99% (95%CI: -4.06 to +2.07)<br><br>**Percentage change in Emotional Eating:**<br>Measured by the Emotional Overeating Questionnaire [74]<br>Post-intervention data not provided (only baseline and follow up).	
Mason *et al.* (2018) [50]<br>USA	**Design:** Single group design<br>**Sample size:** n = 104 (61 completers at post-intervention)<br>**Mean age:** 46.07 years (SD:14.64).<br>**Gender:** Not reported<br>**Ethnicity:** 68.3% White, 4.8% Black, 10.6% Hispanic/Latino, 9.6% Asian/Pacific Islander, 0.0% Native American, 1.0% Declined to answer<br>**Mean weight**: 186.89lbs (84.7Kg) (SD: 13.4)<br>**Mean BMI**: 31.24Kg/m^2 (SD:4.26)	**Interventions:**<br>• Mindfulness<br>**Length of intervention:** 28 days<br>**Setting:** Via a mobile phone<br>**Delivered by:** Video lectures and guidance on Mindfulness practice.	**Percentage weight change:**<br>Mindfulness (n=61) -0.96% (96%CI: -4.55 to 2.63)<br><br>**Percentage change in Emotional Eating:**<br>Measured by the Food Craving Questionnaire-Trait-Reduced (FCQ-T-R) [76]<br>Mindfulness (n=61): -23.29% (95%CI: -34.71 to -11.86)	**Tool:** JBI Quasi-experimetal<br>**Score:** 4<br>**Percentage:** 66.7
Moraes et al. (2021) [51]<br>Brazil	**Design:** RCT<br>**Sample size:** n = 64 in the two intervention groups of interest.<br>**Mean age:** EH: 35.98 years (SD:6.76); IT + CBT: 36.18 years (SD:2.75)	**Interventions:**<br>• Education and Health Group (EH)<br>• Interdisciplinary therapy plus cognitive behavioural therapy (IT + CBT)<br>• Compared to a physical activity program (no elements of EE)	**Percentage weight change:**<br>EH (n=33): -2.89% (95%CI: -7.34 to +1.56))<br>IT + CBT (n=31): -3.32% (95%CI: -7.89 to +1.26)<br><br>**Percentage change in Emotional Eating:**	**Tool:** CASP RCT<br>**Score:** 10<br>**Percentage:** 90.9

	**Gender:** EH: 81.8% Female, 18.2% Male; IT +CBT: 77.4% Female, 22.6% Male.<br>**Ethnicity:** Not reported<br>**Mean weight**: EH: 94.88Kg (SD:10.61); IT + CBT: 92.56Kg (SD:12.16)<br>**Mean BMI**: EH: 35.69Kg/m^2 (SD:2.57); IT + CBT: 35.57Kg/m^2 (SD:2.85)	**Length of interventions**: 30 weeks<br>**Setting:** Group (EH), group and individual (IT + CBT)<br>**Delivered by:** Health professionals	Measured by the Dutch Eating Behaviour Questionnaire (DEBQ) [66]<br>EH (n=33): +15.44% (95%CI: -13.71 to +44.58)<br>IT + CBT (n=31): -48.39% (95%CI: -81.21 to -15.58)	
Niemeier et al. (2012) [52]<br>USA	**Design:** Single group design pilot<br>**Sample size:** n = 21 (18 completers at post-intervention)<br>**Mean age:** 52.2 years (SD:7.6)<br>**Gender:** 90.5% Female, 9.5% male<br>**Ethnicity:** 90% non-Hispanic, 4.8% Hispanic, 4.8% other<br>**Mean weight**: 88.8Kg (SD:11.2)<br>**Mean BMI**: 32.8Kg/m^2 (SD:3.4)	**Interventions:**<br>• Acceptance-Based Behavioural Intervention (ABBI) (BWL +Acceptance based)<br>**Length of interventions:** 24 weeks<br>**Setting: G**roup<br>**Delivered by:** A PhD- level Clinical Psychologist; a Clinical Psychology Intern; and a masters-level nurse with expertise in behavioural weight loss.	**Percentage weight change:**<br>ABBI (n=18): -13.51% (95%CI: -21.67 to -5.36)<br><br>**Percentage change in Emotional Eating:**<br>Measured by the Eating Inventory [69]<br>ABBI (n=18): -43.33% (95%CI: -81.52 to -5.15)	**Tool:** JBI Quasi-experimetal<br>**Score:** 4<br>**Percentage:** 66.7
Paans et al. (2020) [53]<br>MooDFOOD<br>Netherlands	**Design:** RCT<br>**Sample size:** n = 372 for intervention group<br>**Mean age:** 47.8 years (SD:12.6)<br>**Gender:** 78.2% Female<br>**Ethnicity:** Not reported<br>**Mean weight**: Not reported<br>**Mean BMI**: 31.2Kg/m^2 (SD:3.8)	**Interventions:**<br>• Food-related behavioural activation therapy (Behavioural therapy F-BA).<br>Groups:<br>• Multi-nutrient supplement + FBA<br>• Placebo supplement + FBA<br>• Multi-nutrient supplement<br>• Placebo supplement<br>**Length of interventions:** 1-year	**Percentage weight change:**<br>F-BA (n=372): -0.23% 95%CI: -3.02 to 2.56)<br><br>**Percentage change in Emotional Eating:**<br>Measured by the Three Factor Eating Questionnaire (TFEQ) [68]<br>F-BA (n=372): -30.80% (95%CI: -43.53 to -18.07)	**Tool:** CASP RCT<br>**Score:** 10<br>**Percentage:** 90.9

		**Setting:** 15 individual sessions, 6 group sessions<br>**Delivered by:** Psychologists familiar with behavioural activation.		
Palmeira et al. (2017) [54]<br>Kg-free<br>Portugal	**Design:** RCT<br>**Sample size:** n = 27 for intervention group<br>**Mean age:** 41.97 years (SD:8.79).<br>**Gender:** 100% women<br>**Ethnicity:** Not reported<br>**Mean weight**: Not reported<br>**Mean BMI**: 34.82Kg/m² (SD:5.26)	**Interventions:**<br>• Acceptance and Commitment Therapy (ACT)<br>• Compared to treatment as usual controls<br>**Length of interventions**: 12 weeks<br>**Setting:** Group, in person<br>**Delivered by:** A Clinical Psychologist with previous training in contextual-behavioural therapies and a clinical psychology master's student.	**Percentage weight change:**<br>Only BMI reported.<br><br>**Emotional Eating outcome:**<br>Measured by the Three Factor Eating Questionnaire (TFEQ) [68]<br>ACT (n=27): -13.89% (95%CI: -55.06% to 27.28%)	**Tool:** CASP RCT<br>**Score:** 10<br>**Percentage:** 90.9
Rieger et al. (2017) [55]<br>Australia	**Design:** RCT<br>**Sample size:** n = 201 (118 completers post-intervention)<br>**Mean age:** 47.01years (SD:11.52)<br>**Gender:** 73.6% Female<br>**Ethnicity:** Not reported<br>**Mean weight**: 105.58Kg (SD:20.70)<br>**Mean BMI**: 37.71Kg/m² (SD:6.32)	**Interventions:**<br>Cognitive behaviour therapy (CBT):<br>• Weight loss either Alone (CBT-A)<br>• With the addition of a Support Person (CBT- SP).<br>**Length of interventions:** 12 months<br>**Setting:** Group, in person<br>**Delivered by:** Five therapists with postgraduate degrees in clinical psychology.	**Percentage weight change:**<br>CBT A (n=61): -5.30% (95%CI: -10.33 to -0.28)<br>CBT-SP (n=57): -7.53% (95%CI: -13.28 to -1.78)<br><br>**Percentage change in Emotional Eating:**<br>Measured by the Binge Eating Scale [71]<br>CBT-A (n=61): -36.96% (95%CI: -60.01 to -13.92)<br>CBT-SP (n=57): -39.04% (95%CI: -62.95 to -15.12)	**Tool:** CASP RCT<br>**Score:** 10<br>**Percentage:** 90.9
Roosen et al. (2012) [21]<br>Netherlands	**Design:** Pilot, Single group design<br>**Sample size:** n = 35<br>**Mean age:** 39.2 years (SD:11.02)	**Intervention:**<br>• Dialectical Behaviour Therapy (DBT)<br>**Length of intervention:** 20 weeks	**Percentage weight change:**<br>Only BMI reported.	**Tool:** JBI Quasi-experimetal<br>**Score:** 4<br>**Percentage:** 66.7

	**Gender:** 86% Female, 15% Male<br>**Ethnicity:** Not reported<br>**Mean weight**: Not reported<br>**Mean BMI**: 35.42Kg/m² (SD:2.62)	**Setting:** Group, in person<br>**Delivered by:** Two trained co- therapists	**Percentage change in Emotional Eating:**<br>Measured by the Dutch Eating Behaviour Questionnaire (DEBQ) [66]<br>DBT (n=35): -28.57% (95%CI: -69.50 to 12.36)	
Salvo et al. (2021) [56]<br>Brazil	**Design:** Pilot, Single group design, with mixed-methods evaluation<br>**Sample size:** n = 20<br>**Mean age:** 48.15 years (SD:8.57)<br>**Gender:** 100% Female<br>**Ethnicity:** Not reported<br>**Mean weight**: 91.9Kg (SD:19.4)<br>**Mean BMI**: 35.3Kg/m² (SD:6.6)	**Intervention:**<br>• Mindfulness-Based Eating Awareness Training (MB-EAT)<br>**Length of intervention**: 13 weeks<br>**Setting:** Group<br>**Delivered by:** Research Team	**Percentage weight change:**<br>MB-EAT (n=20): -1.96% (95%CI: -6.11 to 2.19)<br><br>**Percentage change in Emotional Eating:**<br>Measured by the Eating Attitudes Test (EAT-26) [73]<br>MB-EAT (n=20): -29.57% (95%CI: -50.23 to -8.90)	**Tool:** JBI Quasi-experimetal<br>**Score:** 5<br>**Percentage:** 83.3
Sampaio et al. (2021) [57]<br>Brazil	**Design:** RCT<br>**Sample size:** n = 27 for intervention group<br>**Mean age:** 49 years (SD:11.0)<br>**Gender:** 100% Female<br>**Ethnicity:** 7.4% White, 40.7% Black, 51.9% Mixed<br>**Mean weight**: Overweight group: 69.3Kg (SD not reported). Obesity group: 92.4Kg (SD not reported).<br>**Mean BMI**: Overweight group: 28.2 Kg/m² (SD not reported). - 35.8Kg/m² (SD not reported).	**Interventions:**<br>• Mediation practice.<br>• Compared to a control group<br>**Length of interventions**: 7 months<br>**Setting:** Group<br>**Delivered by:** A Nutritionist, a family doctor, a Gynecologist, and an Endocrinologist.	**Percentage weight change:**<br>Insufficient data reported to calculate.<br><br>**Percentage change in Emotional Eating:**<br>Measured by the Dutch Eating Behaviour Questionnaire (DEBQ) [66]<br>Mediation practice (n=27): -43.33% (95%CI: -62.40 to -24.26)	**Tool:** CASP RCT<br>**Score:** 11<br>**Percentage:** 100
Spadaro et al. (2017) [58]	**Design:** RCT<br>**Sample size:** n = 46	**Interventions:**	**Percentage weight change:**	**Tool:** CASP RCT<br>**Score:** 10

USA	**Mean age:** 45.2 years (SD:8.2)<br>**Gender:** 87% Female, 13% Male<br>**Ethnicity:** 78.3% Caucasian, 21.7% African American<br>**Mean weight**: SBWP: 93.2Kg (SD:2.8). SBWL + MM: 90.7Kg (SD:2.9)<br>**Mean BMI**: 32.5Kg/m² (SD:3.7)	• Behavioural Weight Loss Program (SBWP)<br>• Behavioural Weight Loss Program + Mindfulness Meditation (SBWP+MM)<br>**Length of intervention**: 6 months<br>**Setting**: Group<br>**Delivered by:** A doctoral student in exercise physiology	SBWP (n=-24): -4.40% (95%CI: -9.64 to 0.85)<br>SBWL + MM (n=22): -7.61% (95%CI: -14.13 to -1.09)<br><br>**Percentage change in Emotional Eating:**<br>Measured by the Eating Inventory [69]<br>SBWP (n=-24): -14.16% (95%CI: -38.36 to 10.04)<br>SBWL + MM (n=22): -28.57 (95%CI: -58.56 to 1.42)	**Percentage:** 90.9
Tham and Chong (2020) [59]<br>Medical & Mind Weight Loss Redefine CBT Programme<br>Australia	**Design:** Single group design<br>**Sample size:** n = 120<br>**Mean age:** Not reported<br>**Gender:** 57.5% Female, 42.5% Male<br>**Ethnicity:** Not reported<br>**Mean weight**: 98.58Kg (SD:22.72)<br>**Mean BMI**: 35.45Kg/m² (SD:4.24)	**Intervention:**<br>• Cognitive Behavioural Therapy (CBT)<br>**Length of intervention:** 26 weeks<br>**Setting:** Online<br>**Delivered by:** The research team through online resources.	**Percentage weight change:**<br>CBT (n=120): -14.69% (95%CI: -22.24 to -7.14)<br><br>**Percentage change in Emotional Eating:**<br>Measured by the Emotional-Eater-Questionnaire [72]<br>CBT (n=120): -56.30% (95%CI: -77.22 to -35.39)	**Tool:** JBI Quasi-experimetal<br>**Score:** 5<br>**Percentage:** 83.3
Thomas et al. (2019) [60]<br>POWER & MOREPOWER<br>USA	**Design:** RCT<br>**Sample size:** n = 51<br>**Mean age:** 57.92 years (SD: 10.04)<br>**Gender:** 100% Female<br>**Ethnicity:** 96% White, 2% Black/African American, 2% Hispanic/Latino<br>**Mean weight**: 94.42Kg (SD:17.75)	**Interventions:**<br>• Exercise and nutrition counselling (BWL) POWER<br>• Exercise and nutrition counselling plus (Mindfulness) MORE + POWER<br>**Length of interventions:** 10 weeks<br>**Setting:** Group, in person	**Percentage weight change:**<br>POWER (n=25): -3.37% (95%CI: -8.02 to 1.27)<br>MORE POWER (n=26): -4.04% (95%CI: -9.15 to 1.08)<br><br>**Percentage change in Emotional Eating:**	**Tool:** CASP RCT<br>**Score:** 10<br>**Percentage:** 90.9

	**Mean BMI**: 34.69Kg/m^2 (SD:7.39)	**Delivered by:** A Registered Dietitian and a master's- level licensed clinical social worker.	Measured by the Dutch Eating Behaviour Questionnaire (DEBQ) [66]<br>POWER (n=25): -0.94% (95%CI: -7.29 to 5.40)<br>MORE POWER (n=26): -16.04% (95%CI: -28.86 to -3.21)	

Supplementary Table S3: Hierarchy of Inclusion and Exclusion Criteria

Criteria	How many papers excluded at each level
Non-English	1
Non-human studies	0
Not published in a peer-reviewed journal	7
Study design is a systematic review/umbrella review/review of reviews/scoping review/study protocol (not primary data collection)	6
Does not involve interventions with components specifically to address EE	118
Participants were not adults aged 18 and above	2
Outcome is not related to Effectiveness (weight loss or control of eating)	21
Less than 70% of sample had a BMI of 25Kg/m^2 or above	11
More than 70% of the sample had a diagnosed eating disorder	49
Medical or pharmacological intervention	22
Participants were post-bariatric surgery	10
Sample size of less than 10 participants	1
Authors did not respond to email requesting the data on weight or EE scores	5
**Total papers excluded**	**253**

Supplementary Table S4: Critical Appraisal Tool For Randomised Control Trials [61]

CASP Critical Appraisal Tool For Randomised Control Trial													
Author, Year	Section A			Section B			Section C			Section D		Total	Score (%)
	Q1	Q2	Q3	Q4	Q5	Q6	Q7	Q8	Q9	Q10	Q11		
Afari et al. (2019)	Y	Y	Y	Y	Y	Y	Y	Y	Y	Y	Y	11	100
Annesi, et al. (2016)	Y	Y	N	N	Y	Y	Y	Y	Y	Y	Y	9	81.8
Annesi, (2019)	Y	Y	Y	N	Y	Y	Y	Y	Y	Y	Y	10	90.9
Bacon et al., (2005)	Y	Y	Y	N	Y	Y	Y	Y	Y	Y	Y	10	90.9
Carbine et al., (2021)	Y	Y	Y	Y	Y	Y	Y	Y	Y	Y	Y	11	100
Carpenter et al. (2019)	Y	Y	Y	Y	Y	Y	Y	Y	Y	Y	Y	11	100
Daubenmier et al. (2016)	Y	Y	Y	Y	Y	Y	Y	Y	Y	Y	Y	11	100
Forman et al., (2013)	Y	Y	N	N	Y	Y	Y	Y	Y	Y	Y	9	81.8
Goldbacher et al., (2016)	Y	Y	Y	Y	Y	Y	Y	Y	Y	Y	Y	11	100
Hepdurgun et al., (2020)	Y	Y	Y	N	Y	Y	Y	Y	Y	Y	Y	10	90.9
Keränen et al (2009)	Y	Y	Y	Y	Y	Y	Y	Y	Y	Y	Y	11	100
Kim et al. (2021)	Y	Y	Y	Y	Y	Y	Y	Y	Y	Y	Y	11	100
Lillis et al., (2016)	Y	Y	Y	Y	Y	Y	Y	N	N	Y	Y	11	100

Malknia-Pykh, (2012)	Y	Y	Y	N	Y	Y	Y	Y	Y	Y	Y	10	90.9
Manzoni et al., (2009)	Y	Y	Y	N	Y	Y	Y	N	Y	Y	Y	9	81.8
Moraes et al., (2021)	Y	Y	Y	N	Y	Y	Y	Y	Y	Y	Y	10	90.9
Paans et al., (2020)	Y	Y	Y	Y	Y	Y	Y	N	Y	Y	Y	10	90.9
Palmeiraet al., (2017)	Y	Y	Y	Y	N	Y	Y	Y	Y	Y	Y	10	90.9
Rieger et al., (2017)	Y	Y	Y	N	Y	Y	Y	Y	Y	Y	Y	10	90.9
Sampaioet al., (2019)	Y	Y	Y	Y	Y	Y	Y	Y	Y	Y	Y	11	100
Spadaro et al., (2017)	Y	Y	N	Y	Y	Y	Y	Y	Y	Y	Y	10	90.9
Thomas et al., (2019)	Y	Y	Y	Y	N	Y	Y	Y	Y	Y	Y	10	90.9

Supplementary Table S5: JBI Checklist for Quasi-Experimental Studies (Non-Randomised Experimental Studies) [62]

JBI Checklist for Quasi-Experimental Studies (Non-Randomised Experimental Studies)											
Author, year	Q1	Q2	Q3	Q4	Q5	Q6	Q7	Q8	Q9	Total	Score (%)
Chung et al., (2016)	Y	N/A	N/A	N	Y	Y	N/A	Y	Y	5/6	83.3
Frayn et al., (2020)	Y	N/A	N/A	N	Y	Y	N/A	Y	Y	5/6	83.3
Hanson et al, (2019)	Y	N/A	N/A	N	Y	Y	N/A	Y	Y	5/6	83.3
Hawkins et al., (2021)	Y	N/A	N/A	N	Y	Y	N/A	Y	Y	5/6	83.3
Hunot-Alexander et al., (2021)	Y	N/A	N/A	N	N	Y	N/A	N	N	2/6	33.3
Kearney et al., (2012)	Y	N/A	N/A	N	Y	Y	N/A	Y	Y	5/6	83.3
Kidd et al, (2013)	Y	N/A	N/A	N	Y	N	N/A	Y	Y	4/6	66.7
Mason et al., (2018)	Y	N/A	N/A	N	Y	N	N/A	Y	Y	4/6	66.7
Niemeier et al., (2012)	Y	N/A	N/A	N	Y	N	N/A	Y	Y	4/6	66.7
Roosen et al., (2012)	Y	N/A	N/A	N	Y	N	N/A	Y	Y	4/6	66.7
Salvo et al., (2021)	Y	N/A	N/A	N	Y	Y	N/A	Y	Y	5/6	83.3
Tham at al., (2020)	Y	N/A	N/A	N	Y	Y	N/A	Y	Y	5/6	83.3
